# CamoEvo: An open access toolbox for artificial camouflage evolution experiments

**DOI:** 10.1111/evo.14476

**Published:** 2022-03-30

**Authors:** George R. A. Hancock, Jolyon Troscianko

**Affiliations:** ^1^ Centre for Ecology and Conservation University of Exeter Penryn TR10 9FE United Kingdom

**Keywords:** CamoEvo, camouflage, evolution, genetic algorithms, optimization, selection

## Abstract

Camouflage research has long shaped our understanding of evolution by natural selection, and elucidating the mechanisms by which camouflage operates remains a key question in visual ecology. However, the vast diversity of color patterns found in animals and their backgrounds, combined with the scope for complex interactions with receiver vision, presents a fundamental challenge for investigating optimal camouflage strategies. Genetic algorithms (GAs) have provided a potential method for accounting for these interactions, but with limited accessibility. Here, we present CamoEvo, an open‐access toolbox for investigating camouflage pattern optimization by using tailored GAs, animal and egg maculation theory, and artificial predation experiments. This system allows for camouflage evolution within the span of just 10–30 generations (∼1–2 min per generation), producing patterns that are both significantly harder to detect and that are optimized to their background. CamoEvo was built in ImageJ to allow for integration with an array of existing open access camouflage analysis tools. We provide guides for editing and adjusting the predation experiment and GA as well as an example experiment. The speed and flexibility of this toolbox makes it adaptable for a wide range of computer‐based phenotype optimization experiments.

Camouflage has long served as an example of the adaptive value of animal coloration, allowing animals to avoid predation by impeding detection or recognition (Thayer et al. 1918; Endler 1981; Cuthill 2019). Camouflage strategies, such as crypsis, are dependent on the interaction between an animal and its background, so the structure and composition of natural backgrounds influences the evolution and appearance of camouflage patterns. Camouflage strategies, such as crypsis, are dependent on the interaction between an animal and its background, so the structure and composition of natural backgrounds influences the evolution and appearance of camouflage patterns (Endler 1981; Caro 2005). For the past 20 years, psychophysics experiments in the field, lab, and online have been used to investigate how different aspects of animal patterns (luminance, color, pattern, edge‐disruption, edge‐enhancement, countershading) affect the camouflage of target objects/animals against different backgrounds (Stevens and Merilaita 2008; Rowland et al. 2008; Egan et al. 2016; Michalis et al. 2017; Troscianko et al. 2017a). However, these experiments have been typically confined to manipulating or measuring a limited number of camouflage parameters due to the almost limitless phenotypic space of animal patterns, and potential for complex interactions between camouflage strategies, background appearance, and predator behavior.

Genetic algorithms (GAs) present a potential solution to this problem by allowing large combinations of phenotypes to be tested and then improved upon using evolutionary computing inspired by natural selection (Fig. 1) (Mitchell 1996; Hamblin 2013). GAs have been widely used since their development during the mid‐1970s as an optimization tool for problems that have multiple solutions and expansive parameter spaces (Holland 1975; Goldberg and Holland 1988; Whitley 1994). Despite this, GAs have been infrequently used within camouflage ecology research, having only been used for experiments on camouflage polymorphism, generalist‐specialist strategies, and artificial selection of textural camouflage (Bond and Kamil 2002; Merilaita 2003; Sherratt et al. 2007; Reynolds 2011). Previous studies have relied upon complex custom‐written evolutionary frameworks, large sample sizes, and long evolutionary times (Reeves 1993; Zhai et al. 1996; Talas et al. 2020; Fennell et al. 2021). Advances in GAs have allowed smaller populations to be more effective at exploring optimization and for faster optimization in general, by using adaptive mutation rates, specialized mutations, and polygamous mating systems (Reeves 1993; Marsili Libelli and Alba 2000; Kumar 2012; Soni and Kumar 2014). These features can also be used for improving the performance of GAs used in camouflage optimization and for testing hypotheses for the interactions of selection and life history on camouflage evolution, though we will focus on the former for this paper.

**Figure 1:**
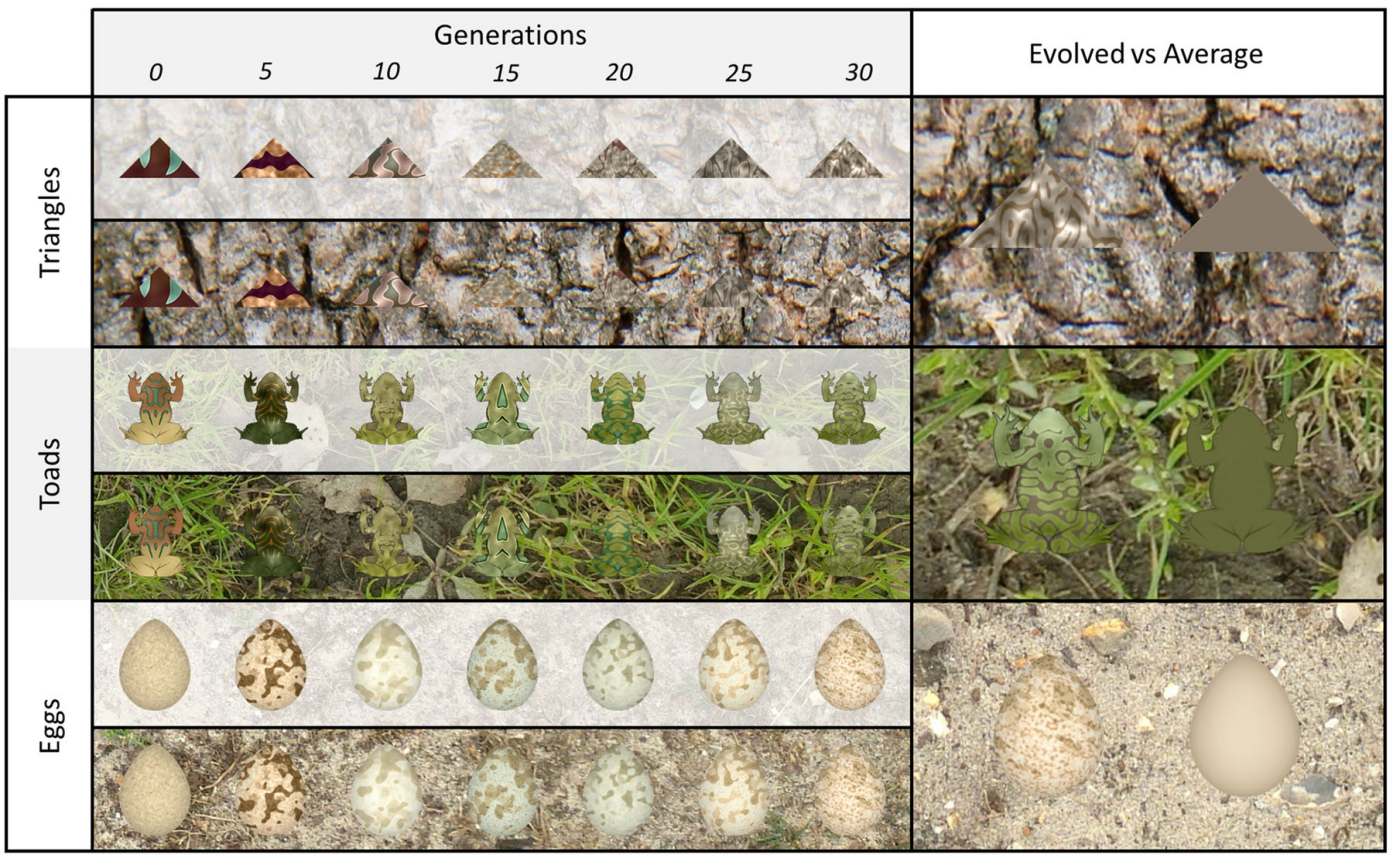
Example evolutionary lines from CamoEvo with different prey shapes (Triangle, Toad, and Eggs) against different backgrounds (Tree Bark, Bog Grass, and Eroded Scrub). On the left is the fittest (longest to find) individual from every five generations, for 30 generations, against the background they were evolved against. Above shows the target without and below with the background. On the right is the best target compared with a target that matches the global average CIELAB values for the background.

Here, we present the CamoEvo Toolbox for ImageJ (Schneider et al. 2012). This toolbox consists of three distinct components: (i) a customizable decimal‐based GA designed for evolving populations of computer‐generated images, dubbed ImageGA; (ii) a decimal‐gene determined pattern generation systems for animals and eggs (Fig. 1); and (iii) a user‐friendly visual search‐based psychophysics experiment that uses ImageGA to optimize patterns for camouflage against assigned backgrounds, though it can also be used for conspicuousness (Fig. 2). CamoEvo is designed to be advanced, customizable, and user friendly, acting as a tool for animal color research.(Bonney et al. 2014; Troscianko et al. 2017b; Niu et al. 2021). By using ImageJ, CamoEvo and its GA can easily be integrated with a variety of image generation and analysis tools, in addition to other open source plugins and toolboxes such as MICA, Acuity View, and QCPA (Troscianko and Stevens 2015; Caves and Johnsen 2018; van den Berg et al. 2020). Here, after we discuss the functionality and methodology of CamoEvo, an example experiment and a list of possible avenues of research are given.

**Figure 2:**
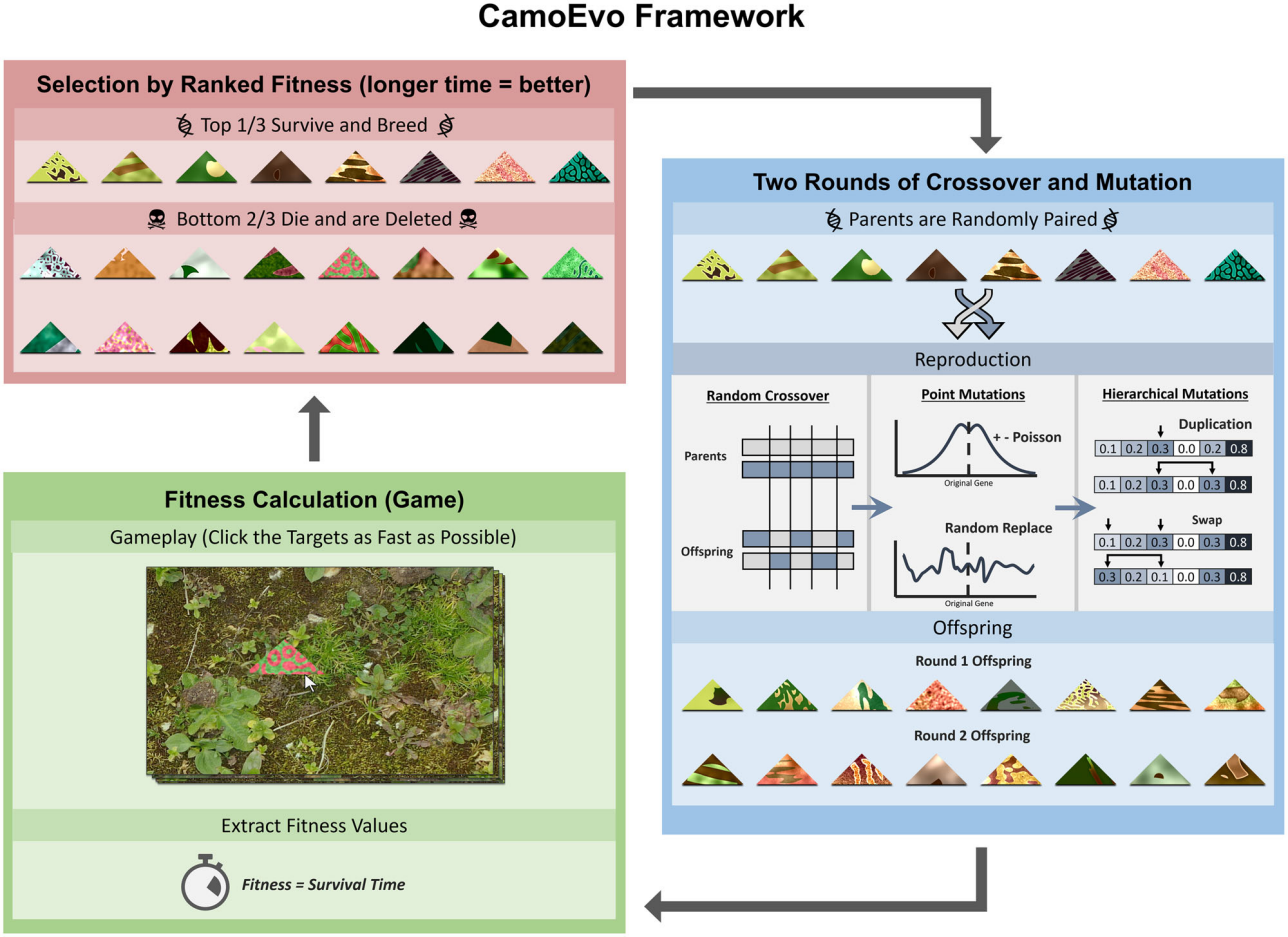
Schematic outline of an example run of CamoEvo using the default genetic algorithm settings. The game generates fitness values (survival time). The survival values are then passed on to the genetic algorithm (ImageGA) that categorizes them by rank for breeding (highest) or deletion (lowest). Offspring are then created through random mating and crossover between survivors and subsequent random mutations, such as addition/subtraction of Poisson distributed noise or duplication of sections of the genome mimicking biological systems. A new population then consists of the unaltered parents from the prior population and their mutant offspring. These are then fed back into the game in a loop, until the assigned number of generations has been completed.

**CamoEvo Example**

**Description**: A showcase of CamoEvo creating effective camouflage within the span of 15 generations against three different backgrounds (heathland scrub, leaf litter, and spring vegetation) using three different starting populations

**Hypotheses**: Fitness should improve with evolutionary time (Generation) along with known camouflage measures.

**Methods**: Using CamoEvo, a population of *n* = 24 triangle targets were evolved over the course of 15 generations against natural habitats photographed with an ASUS A002 smartphone and calibrated using a 5% gray reflectance standard. The game was run using CamoEvo's default genetic algorithm settings and the stimuli consisted of triangle targets (150 px, 75 px) shown against one of 24 cropped images (1478 px, 1130 px) from each habitat. On a typical laptop display of size 30, 16 cm, the target is 2, 1 cm. Targets were allowed to occupy a CIELAB space with a luminance range of 0, 100, A (green‐red) range of −60, 60, and B (blue‐yellow) range of −10, 70. For each generation, CamoEvo automatically generated a set of stimuli consisting of 24 slides. To construct each slide, CamoEvo chooses one of the targets, using a random sequence, and copies it to a random location on the background image. For each slide, CamoEvo measured the mean and standard deviation of the L (luminance), A (green‐red), and B (blue‐yellow) channels for both the target and its local background (circle of diameter = 2× target diameter). The difference between the mean L, A, and B values for the target and background (e.g., difference in L mean = ΔμL) and the difference in standard deviation (e.g., difference in L StDev = ΔσL) were then calculated. Additionally, the disruption of the targets edge by its pattern was measured for each channel using the “GabRat” method, which uses Gabor filters to compare the intensity ratio between the perceived (“false”) and actual edges of the object (McCamy 1992; Troscianko et al. 2016; Renoult et al. 2017; Troscianko et al. 2017a). These measures were chosen as they are common metrics for assessing the level of camouflage.

Volunteers were recruited from the University of Exeter (aged 20–30), were randomly assigned one of the nine background treatments, and were given 1 h to complete the experiment on their own computer. This was done both as a test of system compatibility and due to COVID‐19 restrictions. Computer monitors, operating system, and viewing distance were not standardized. Before each slide started, the volunteers were instructed by CamoEvo to position their cursor at the center of the screen and to only move it after seeing the target, which they then had to click on as quickly as possible within a span of 15 s per slide. After each generation was completed, a new population was automatically generated. Ranked survival time was used as the measure of fitness. Prior work shows the first target typically takes far longer to find than subsequent ones (Troscianko et al. 2018). To control for this effect, an additional two targets were shown at the beginning of the game. These were randomly generated within the phenotype space and were not included in the population.

**Statistics**: All statistics were performed using R version 4.1.2 and mixed models were created with LME4 1.1‐27.1 (R Core Team 2021). Models were generated for all recorded camouflage measures. As the influence of Generation is likely to plateau the closer the population is to a global or local optima, we fitted generation with a polynomial. The background was included as a fixed effect and the player. Each model was checked for normality and homogeneity of variance. An example model for the effect of generation and background on mean L difference is as follows:

Lmer(LocalDifMeanL∼Generation×Background+(1|player).

We also measured the correlation between survival time and the other camouflage metrics to determine whether they influenced the fitness values. To do this, we used generation and the display sequence of the targets as additional random effects and survival time as the response variable (see Supporting Information). An example model for the effect of L difference and background on survival time is as follows:

$\begin{array}{c} lmer(log(Survival\_Time)∼LocalDifMeanL\times Background\\ +(1|Generation)+(1|player)+(1|slide\_order). \end{array}$

All statistics were performed using R version 4.1.2 and mixed models were created with LME4 1.1‐27.1 (R Core Team 2021).

**Results**: As predicted, the survival time (fitness) significantly increased across all three background treatments, though at a significantly slower rate for vegetation compared with the other habitat treatments (Table 1). ΔμL, ΔμA, and ΔμB decrease for all three habitats, though ΔμB decreases faster for leaflitter and ΔμB decreases slower for scrubland compared to vegetation. ΔσL decreased significantly for leaf litter and ΔσA decreased significantly for scrubland, otherwise contrast match actually increased slightly, but not significantly, for the remaining combinations. GabRatL increases, with a faster increase for scrubland compared to the other habitats. However, only scrubland and leaf litter increase in GabRatA and only leaf litter increases in GabRatB. So, although the vegetation background does not improve as much fitness wise, it does improve for a number of camouflage metrics. The R scripts used to run the analyses, the original background images, and the evolved phenotypes can all be found within the Supporting Information. Plots of some of the camouflage metrics can be found in Figure 3.

**Table 1:** Comparison of the effect of generation and habitat on the camouflage metrics. Estimate, SE, and *P*‐values are shown for polynomial 1 (P1) and below polynomial 2 (P2). Significant *P*‐values are highlighted in green. Significance thresholds were calculated using Bonferroni correction for two pair‐wise comparisons (threshold, *P* < 0.005)

	Leaf Litter:Generation (Base Value)			Scrubland:Generation vs. Leaf Litter:Generation			Vegetation:Generation vs. Leaf Litter:Generation		
Variable	*β*	SE	*P*‐value	*β*	SE	*P*‐value	*β*	SE	*P*‐value
Survival Time Milliseconds (P1)	13.57	1.134	1.57e^−08^	1.83	1.60	0.25	–8.89	1.60	3.25e^−08^
(P2)	0.66	0.18	<2e^−16^	–5.68	1.60	0.00041	–0.76	1.60	0.64
Mean Luminance Difference (P1)	–191.99	16.36	<2e–^16^	–54.4697	23.1357	0.0186	–33.4276	23.1357	0.1486
(P2)	–37.92	16.36	0.0205	102.7461	23.1357	9.23^e–06^	55.2076	23.1357	0.0171
Mean Green‐Red Difference (P1)	–177.79	16.71	<2e^–16^	10.17	23.63	0.67	58.4666	23.63	0.013
(P2)	31.29	16.71	0.061	29.33	23.63	0.21	–8.2781	23.63	0.73
Mean Blue‐Yellow Difference (P1)	–167.06	11.85	<2e^−166^	122.97	16.76	2.73e^−13^	60.19	16.76	0.00033
(P2)	–8.18	11.85	0.49	24.44	16.76	0.14	4.69	16.76	0.78
StDev Luminance Difference (P1)	–33.33	6.98	1.87e^−06^	34.36	9.87	0.00051	38.19	9.87	0.00011
(P2)	–26.54	6.98	0.00015	37.28	9.87	0.00016	25.80	9.87	0.0090
StDev Green‐Red Difference (P1)	–5.68	8.54	0.51	–34.81	12.10	0.0039	7.81	12.10	0.52
(P2)	–10.21	8.54	0.23	35.66	12.10	0.00031	1.35	12.10	0.91
StDev Blue‐Yellow Difference (P1)	–11.45	5.94	0.054	40.64	8.39	1.34e^−06^	13.38	8.39	0.11
(P2)	25.04	5.94	2.51e^−05^	–21.12	8.39	0.012	–22.11	8.39	0.0085
GabRat Luminance (P1)	8.98e^−01^	1.11e^–01^	1.07e^–15^	5.03e^−01^	1.58e^−01^	0.0014	1.34e^−01^	1.58e^−01^	0.39
(P2)	2.52e^−01^	1.11e^–01^	0.024	–6.85e^–01^	1.58e^−01^	1.43e^–05^	–8.01e^–02^	1.58e^−01^	0.61
GabRat Green‐Red (P1)	7.97e^−01^	1.47e^–02^	1.29e^−05^	2.66e^−01^	1.76e^−01^	0.1314	–7.28e^−01^	1.76e^−01^	3.63e^−05^
(P2)	–7.70e^−02^	1.47e^–02^	1.78e^−10^	2.61e^−02^	1.76e^−01^	0.8822	3.23e^−01^	1.76e^−01^	0.0667
GabRat Blue‐Yellow (P1)	1.11	0.12	<2e^−16^	–1.02	0.16	4.98e^−10^	–0.57	0.16	0.00051
(P2)	0.36	0.12	0.0021	–0.40	0.16	0.016	–0.26	0.16	0.12

**Figure 3:**
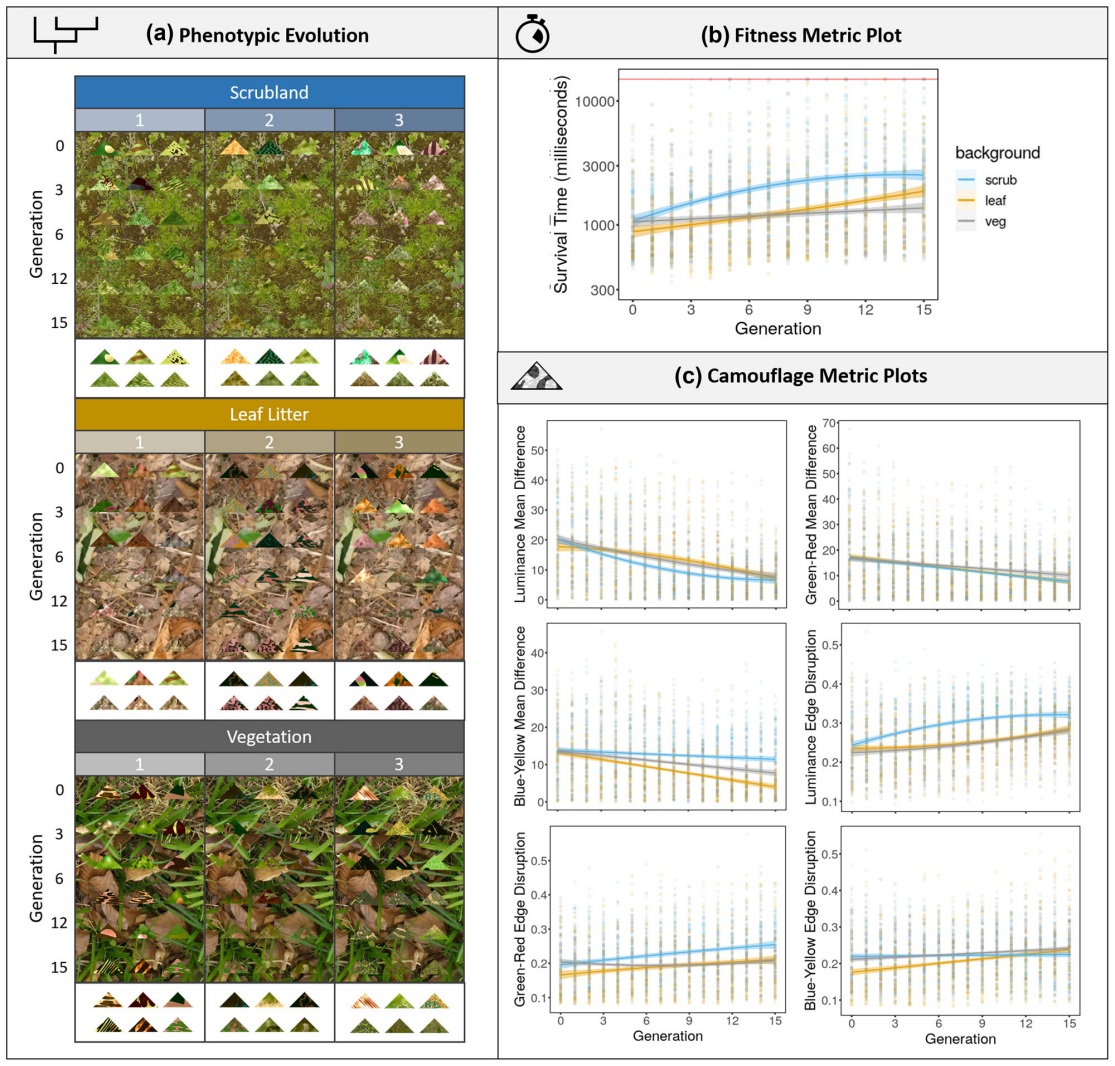
Example data output from CamoEvo for triangle targets evolved against three different backgrounds (Scrubland, Leaf Litter, and Spring Vegetation) using three different starting populations (1, 2, and 3), giving a total of nine treatments. (a) The change in phenotype for the treatments, showing the three highest ranking individuals for every three generations of each population. The first and last generation are shown again at the bottom of each table, against a white background. All phenotypes can be found in the Supporting Information. (b) The change in the fitness measure, log (survival time), for each background; the red line indicates the time‐out time (15,000 ms). (c) The change in the CIELAB camouflage metric for mean L (luminace), A (green‐red), and B (blue‐yellow) difference from the background, as well as the GabRat L, A, and B of the targets. The trend line is shown as a polynomial if polynomial relationship was significant, else it is shown as a linear equation.

## CamoEvo Overview

The following following is a short overview of the CamoEvo toolbox. Detailed instructions are found within the guide book. CamoEvo was designed for ImageJ and the latest version can be downloaded separately or prepackaged with ImageJ, for Mac, Windows, and Linux, found on our GitHub. CamoEvo requires the MICA toolbox to run its edge disruption analyses and this toolbox is included as part of the download (Troscianko and Stevens 2015; Troscianko et al. 2017a).

### PATTERN GENERATION

#### Animal patterns

**Figure 4:**
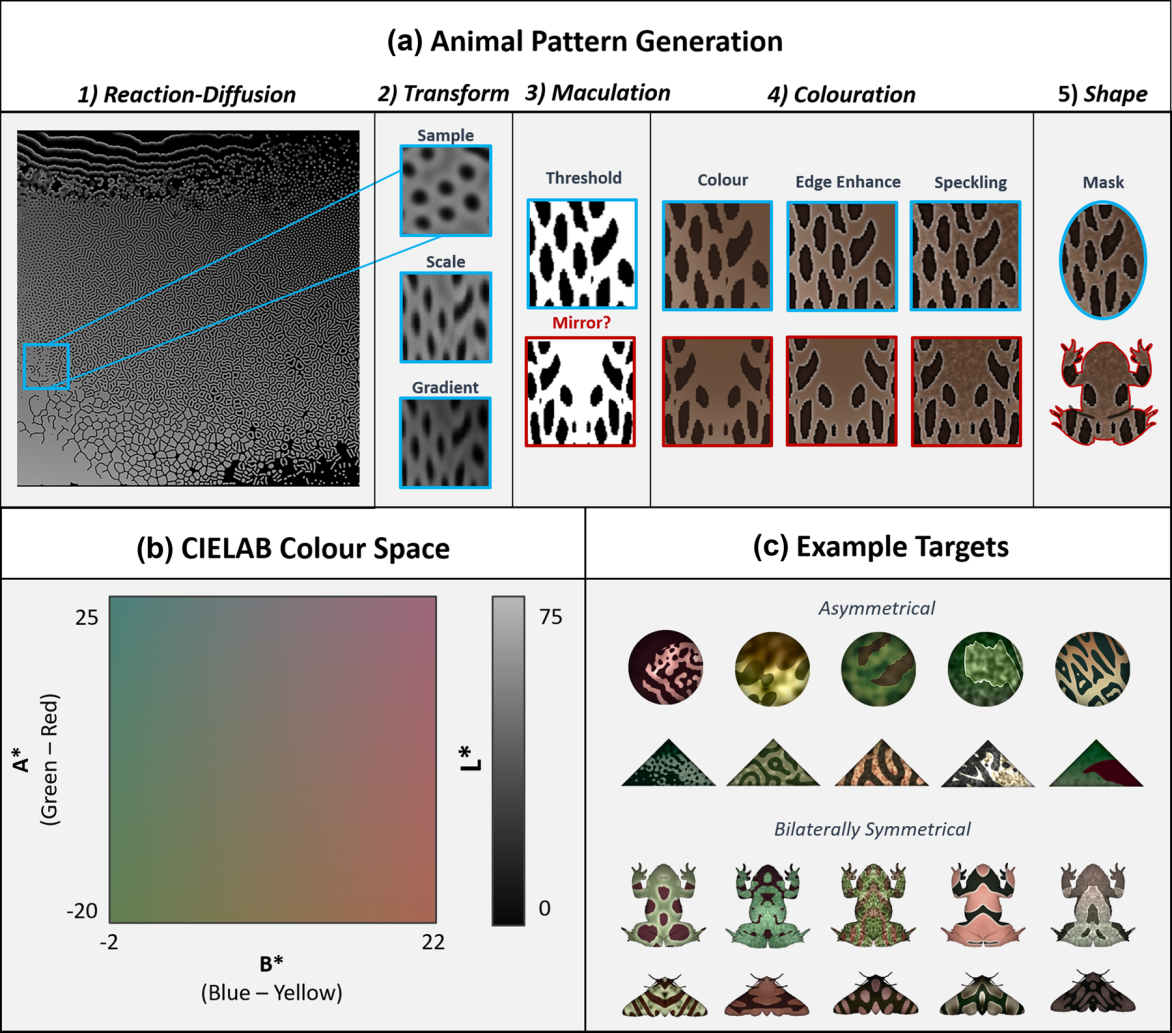
Schematic of the animal pattern generation system used by CamoEvo. (a) A section of the reaction‐diffusion gamut is selected, scaled, and shaded with a gradient before being converted to a binary image (white = maculation, black = background). If specified that the target is symmetrical, then the image is mirrored bilaterally. Coloration is then applied to both the background and the pattern in addition to edge enhancement and speckling. All these features are regulated by different genes. (b) The min and max L, A, and B values for the targets are adjustable allowing for highly salient colorations (e.g., bright blue/purple) to be ignored. (c) Examples of patterns generated within the displayed color ranges are shown.

The patterns of animals are largely made during embryo or integument development and are produced by the migration/deposition of pigments or the creation of structural colors (San‐Jose and Roulin 2017; Orteu and Jiggins 2020). CamoEvo generates animal maculation by sampling from a gamut of Grey‐Scott reaction‐diffusion models, and random noise with Gaussian blurring is used to create speckling (Pearson 1993; Kondo 2002; Allen et al. 2010) (Fig. 4). Striped patterns are generated either from part of the reaction‐diffusion space or by stretching patterns along the *Y*‐axis, akin to Allen et al. (2010). Color is generated using the CIELAB color space as targets within CamoEvo are primarily designed for human detection and it allows for the independent optimization of luminance and color opponent channels (McCamy 1992; Renoult et al. 2017). The color space of the targets can be tailored by adjusting the L (luminance), A (green‐red), and B (blue‐yellow) ranges of just the starting population or of the exploration space. This can be used to limit exploration to the color ranges of a particular clade, background, or observer visual system (e.g., achromatic or dichromatic) (Troscianko et al. 2017b). Targets can be specified as asymmetrical (e.g., circle, triangle) or bilaterally symmetrical (frog, moth; Fig. 4). Edge enhancement is added by increasing the luminance contrast at the edge of the maculation (dark gets darker, light gets lighter), with the intensity, expansion, and Gaussian sigma of the light and dark regions being independently regulated separately between the light and dark (Egan et al. 2016; Sharman and Lovell 2019).

#### Egg patterns

**Figure 5:**
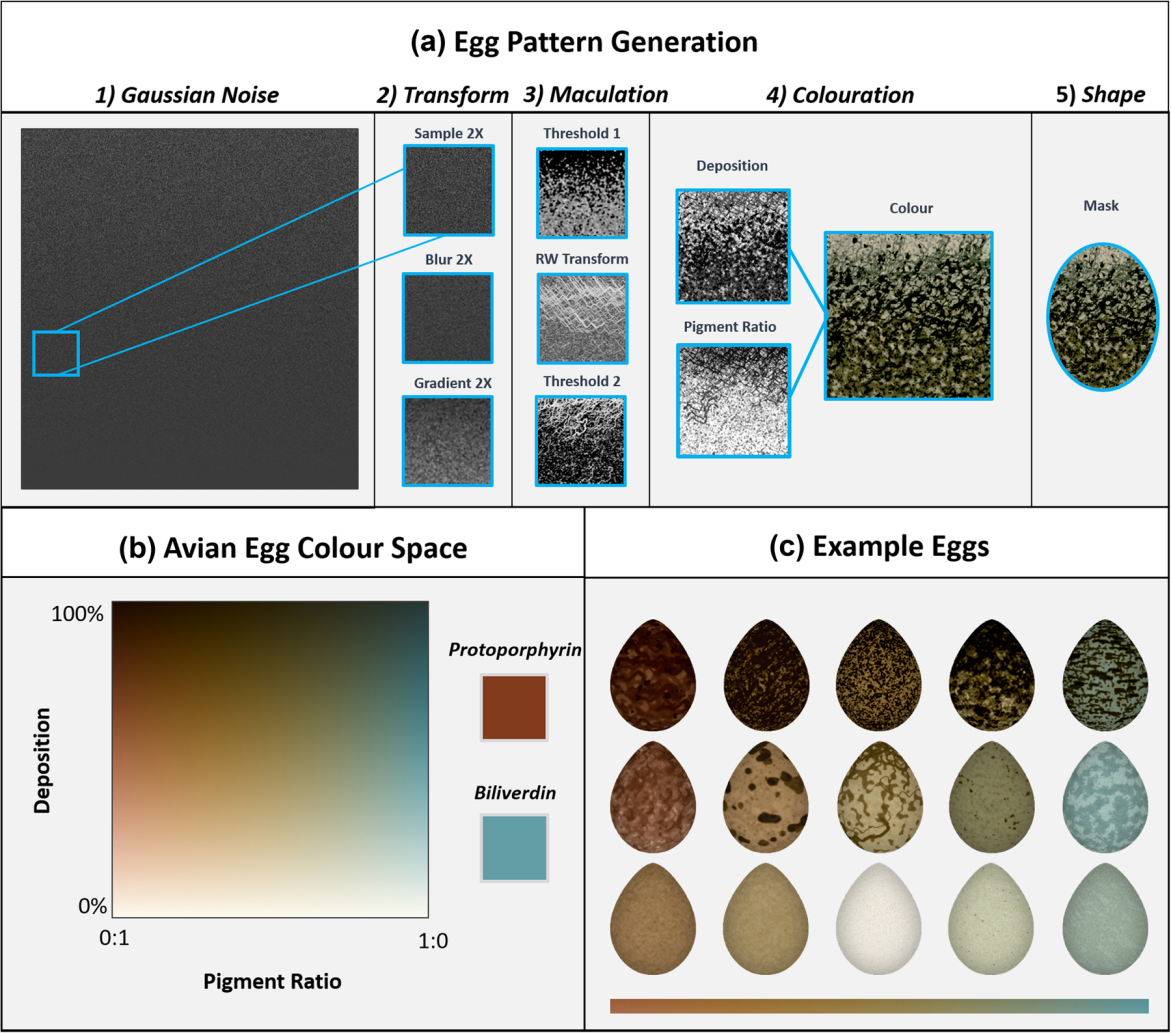
Schematic of the egg pattern generation system used by CamoEvo. (a) A section of a Gaussian noise gradient (*y* = Gaussian StDev) is selected, scaled, and shaded with a gradient; values bellow a threshold are then set to zero. This is repeated twice creating two different patterns, though for one of the patterns random walk is applied by copying and pasting the maculation. The deposition and pigment ratio is calculated for the whole egg by combining the background and the two patterns. (b) Coloration is determined by the interaction between deposition and pigment ratio; the range of pigment ratios can be altered narrowing the color range. (c) Examples of egg patterns generated within the full avian color space and displayed approximately by average pigment ratio (*x*) and deposition (*y*).

Egg patterning is not cell mediated and is not thought to use reaction‐diffusion‐based developmental processes. We created an egg‐specific pattern generator based on existing egg maculation and color theory (Hanley et al. 2015; Pike 2015; Canniff et al. 2018). Maculation is generated by a combination of thresholding (selection of values in a given range), Gaussian noise, Gaussian blurring, and random walk to create splodges, speckles, and spirals found in bird eggs (Fig. 5) (Pike 2015). Coloration is calculated from two dimensions, deposition (amount of pigment) and ratio (biliverdin:protoporphyrin), that are converted into CIELAB values, based on values recorded by Wisocki et al. (2020). The more pigment deposited, the darker the egg and the more saturated the color. However, the color space is restricted to the known bounds of the avian egg coloration (Hanley et al. 2015; Wisocki et al. 2020), though CamoEvo works on the presumption that the backgrounds are not standardized to the same lighting environment as the measurement of the avian eggs and so includes a gene that shifts the luminance of the entire egg independently of deposition, mimicking variation in camera exposure and noise in the correlation between luminance and egg coloration.

### GENETIC ALGORITHM (ImageGA)

ImageGA is a customizable GA designed specifically for the evolution of computer‐generated images. Although there are numerous GA systems available for open access and subscription platforms such as python and MATLAB (Chipperfield and Fleming 1995; Fortin et al. 2012; Kim and Yoo 2019), ImageGA was developed so that all processes of CamoEvo can be run within the same platform, ImageJ, and to provide operators specifically tailored to camouflage optimization. Included within ImageGA are demos for travelling salesperson and color optimization (color at different spatial scales; Jünger et al. 1995). An important concept in camouflage evolution (both biological and in silica) is that although there may be many shifting local fitness optima in the phenotypic landscape, we would not expect a singular “optimum” phenotype to exist. This is because predator learning is expected to exert negative frequency‐dependent selection (Bond and Kamil 2002; Merilaita 2003), and random spawn locations alter the recent evolutionary history (effectively shifting the landscape subtly). As such, maintaining diversity, avoiding fixation, and homing in on these local adaptive optima are key principles that governed all aspects of the design of ImageGA and CamoEvo.

#### Starting population

Populations consist of *N* individuals with decimal‐based genes. To prevent premature clustering and fixation within the population, CamoEvo uses starting populations that are overdispersed. Each gene in the initial population has a uniform distribution with an interval equal to 1/*N* + 1, where *N* equals the population size. This ensures that the initial genotypic space is as wide as possible for the population size (Reeves 1993). However, experimenters can change it to use random, Gaussian distributed, or custom starting populations. The population size is fixed and does not change generation to generation. CamoEvo has been specifically tailored to use small populations (default 24) that are optimized for psychophysics fitness tests, that is, allowing a single individual to sample the entire population before fatigue, and allowing for short run times. However, small populations can exacerbate noise, fixation, and epistasis (suppression of genes by gene interaction) within GAs. To combat these issues, the selection, crossover, and mutation operators have each been tailored in ways that we describe next.

#### Selection & crossover

The number of individuals that get to breed, survive, and/or mutate is adjustable, like many other CamoEvo parameters. By deleting more individuals, the intensity of selection is higher but so too is the rate of diversity loss and fixation (Jebari and Madiafi 2013). Fitness measurements in prey‐search psychophysics experiments will also be subject to a wide range of both environmental (target location, background variation) and receiver noise (reaction‐time, naivety, visual acuity, color perception, cursor/receiver location, and fatigue). CamoEvo uses a mating scheme where the bottom two‐thirds (least fit) of the population die and the remaining one‐third get to survive and breed creating overlapping generations. As the number breeding is less than the number killed, the breeders mate twice producing two rounds of offspring (Kumar 2012). This allows for more recombination from a narrower selection taking advantage of crossover's faster exploration then blind mutation. In both instances of mating, parents are randomly assigned to one another within the breeding pool and so the parents can be promiscuous. As the narrow breeding pool increases the risk of fitter phenotypes being deleted due to random noise, a rescue system is used. The top three (adjustable) individuals in a generation are given a lifeline, where if in the following generation they are placed in the deletion pool, they will instead replace one of the generated offspring rather then re‐adjusting the rankings of the survivors. This mirrors Boltzmann's selection of breeding pairs and protects these individuals from accidental deletion (Jebari and Madiafi 2013; Katoch et al. 2021).

Crossover of genes between mating pairs is determined randomly. Given that the traits of CamoEvo are simplified into decimal values, as opposed to an array of binary values, hybridization of traits would normally not be able to create intermediates. For example, a white parent and a black parent would never make a gray offspring, only another black or white one, as the L value is controlled by one gene. To combat this, CamoEvo uses incomplete crossover where the genes inherited are not identical to those of the parents but instead randomly weighted averages of the parents’ values. This helps to increase exploration and prevent fixation by not limiting the offspring to the same values of the parents. Also using random crossover, as opposed to one‐point or two‐point, breaks up genes that are clustered by location within the chromosome. This can help combat epistasis triggered by localized interacting genes but sacrificing the benefits of preserving clusters.

#### Mutation

To prevent the population from total fixation, ImageGA uses a host of mutation operators that target individual genes (point mutation) or hierarchical clusters of genes. Mutation rate can be set to automatically adjust adaptively based on the genetic diversity, population fitness, and/or the fitness of each individual (Derigs et al. 1999; Marsili Libelli and Alba 2000). Adaptive mutation rates can reduce the loss of genetic diversity and fixation that occurs with each generation can be countered without interfering as much with exploration from crossover in the early generations. By having a mixture of large and small mutations, the GA can explore local optima while also jumping to new potential optima.

Each gene possesses a hierarchical three tag label, for example, “col_mac_lum,” allowing genes to be grouped not just by location on the chromosome but by functions such as coloration, pattern, and edge enhancement. This structure allows for additional types of mutation to occur beyond the standard Poisson/Gaussian or random replacement mutations (Holland 1992). These include the scramble, swap, and duplication mutations that are used to resolve problems where reordering or repetitions of the same or similar solutions allow for faster optimization, as is the case with travelling salesperson problems (Soni and Kumar 2014). Typically, these mutations negatively impact fitness when applied to nonlinked genes as their decimal values do not equate to the same phenotypic values. By labeling the genome, color genes will only copy to their equivalent color genes (e.g., maculation color to pattern color) and likewise for other linked genes, allowing these mutations to be more effectively integrated with the multidimensional problem of pattern optimization and without the need for multiple chromosomes (Cavill et al. 2005; Yang 2014; Tsai et al. 2015). This system can also help to combat epistasis, as epistasis is the result of multiple interacting genes (Hamblin 2013). For example, suboptimal colors can suppress patterns and vice versa. The probability of this occurring is kept low and duplication is carried out using weighted averages to prevent duplication from destroying color contrast or creating false optimums where similarity in characteristics is favored over disparity by the algorithm rather than selection.

### PSYCHOPHYSICS GAME

The visual search task (game) used by CamoEvo to determine fitness has a number of customizable features. By default, the game randomly combines the generated patterns with a list of background images, then tasks the user with clicking on targets as soon as they see them. The experimenter can edit most of its features including the background crop dimensions, target size, time per slide, slide transition method, ranking method, number of targets per slide (one to six targets), and capture method. Stimuli are constructed by cropping the assigned images to 1478, 1130 px, from the image center, with one target placed per slide. Using the default settings, between each slide the user is tasked with positioning their cursor at the center of the screen and to not move their cursor until the target is first spotted (compatible with touch screens). Then for each slide, the time taken for the user to move their mouse (response) and to click on the target (capture) is recorded. Either of these can be used as the fitness ranking for the targets, The default method, however, is a hybrid method, where the response time is used unless the capture time – response time > 600 ms, this is dubbed, Survival Time. This method was created to mitigate the differences in capture time generated by the travel time of the cursor, and the positing of targets near the edge of the screen, both of which are major determinants of capture time (Troscianko et al. 2017b). Mitigating this noise is not just important for measuring the effects of camouflage on detection but also for preventing mis‐ranking of individual targets by the GA. Alternatively, the experimenter can just use response time, capture time, or a location‐based method where left or right mouse clicks indicate whether the target is on the left‐ or right‐hand sides of the screen, respectively (Fennell et al. 2021). The latter method can result in false positives and false negatives from mis‐clicking/guessing. Fitness ranking can be reversed to instead select for conspicuousness as opposed to camouflage.

Another source of noise is that of the receiver, as in psychophysics experiment individuals will vary in their reaction‐time, naivety, visual acuity, color perception, and fatigue. Indeed, participant ID is typically one of the largest sources of variance in camouflage experiments (Troscianko et al. 2018), and this noise can also impact the GAs ranking. The easiest and most common method to minimize this issue is to use one participant either per generation or per population. That way, all fitness values are ranked by the performance of the same individual. However, using one individual will also influence the evolution of the targets due to predator learning. With each generation, the observer's ability to locate the targets is likely to improve and they will develop a search image for the targets (Lawrence and Allen 1983; Troscianko et al. 2018; Troscianko et al. 2021). Alternating observers between generations can offset this providing that each player is naïve (has not played before). Regardless, the rate of evolution is, typically, fast enough that each participant will demonstrate observable selection and camouflage optimization in a population (see Box 1). However, factors such as population structure (pattern diversity, size, and grouping) and background composition (variation within and between images) will still influence fitness and the stability of the targets evolved.

### OUTPUT METRICS

CamoEvo saves all generated target images, coordinates, target orientations, and backgrounds for each play of the game (Box 1). This allows for post hoc image analyses for any stage in the population's evolution. The chromosomes (array of genes) for each population and generation are saved as a .txt file, and can be used for quantifying genotypic selection (e.g., quantifying selection pressure on specific genes) as an alternative to phenotypic selection. When running the default experiment, CamoEvo uses ImageJ and the MICA toolbox tools to measure elements of the background. Calculating the mean and standard deviation for the targets and the surrounding local background (a circle with a diameter two times the target maximum diameter and with the target excluded). In addition to measuring the conspicuousness of the perceived versus the actual target edge (edge disruption) using the “GabRat” measure (Troscianko et al. 2017a). These measures were chosen as they are common quick measures for object camouflage that have been shown to have strong correlation with human and nonhuman detection (Troscianko et al. 2016; Troscianko et al. 2017a; Ramírez‐Delgado and Cueva del Castillo 2020).

## Experimental Design

**Figure 6:**
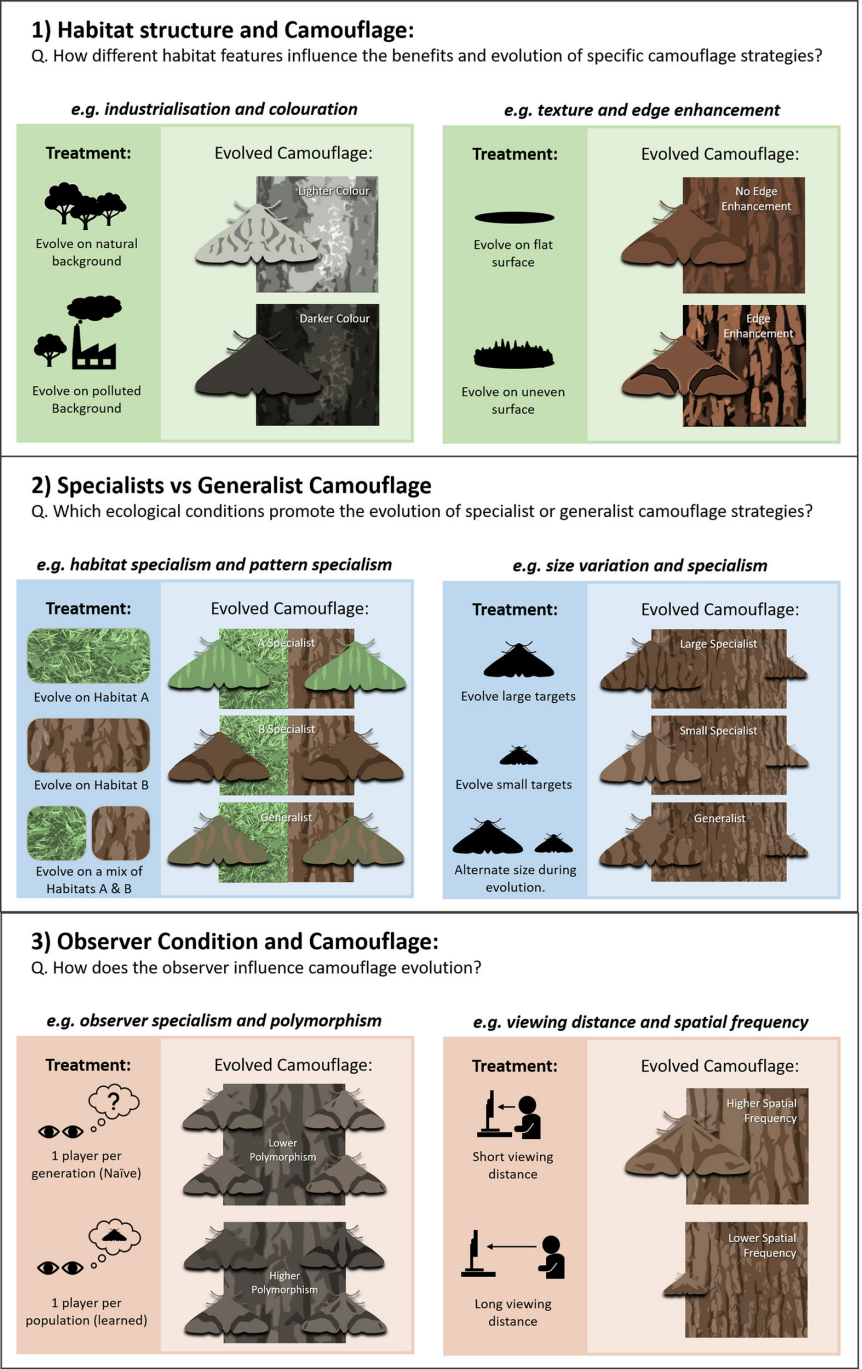
Illustration of hypotheses that are testable using the CamoEvo toolbox, fitted to three areas of current camouflage research. Each experiment only requires the modification of one of the following parameters: backgrounds images, target size, observer used, and observer distance.

CamoEvo can be used for testing a wide range of hypotheses using three main experimental designs: (i) the backgrounds that populations evolve against, (ii) the target appearance (shape, size, and color space), and (iii) the observer individual and their condition (e.g., viewing distance) for each population and generation. Examples of background treatments could include anthropogenic modification, natural clines in habitat structure and composition, or different viewing distances or angles (Cook and Saccheri 2013; Barnett et al. 2017). Different target shapes can be imported using masks (black and white images) and the size can be edited. Observers, of varying naivety, location, or species can be used to drive the evolution of populations (Troscianko et al. 2018). These factors can be manipulated for experiments covering a wide range of different sectors of camouflage research that cannot be easily resolved using standard experimental design (Fig. 6). As the patterns chosen by experimenters may otherwise be biased, what a generalist strategy would look like may be unknown and experimental evolutionary arms races from predator cognition require a GA or GAN(Generative Adversarial Network) to generate the phenotypes for the experiment (Talas et al. 2020; Fennell et al. 2021).

The GA itself can also be altered as part of the experimental design. For example, testing whether mating system influences the speed of camouflage evolution in habitats with differing structure? Where an intermediate strategy is costly (two distinct background patches), assortative mating might be favorable compared to a disassortative or random mating system. If a species/target has a highly specialized predator (narrower search image), mating systems that promote increased polymorphism might be favorable (Lawrence and Allen 1983).

Hypotheses can be tested either by measuring and comparing camouflage phenotypes using image analysis (Box 1) or by measuring the intensity of selection (rate of change in mean or variance) acting on the genes of interest (maculation, color, and edge‐enhancement) (Lande and Arnold 1983; Arnold and Wade 1984; Troscianko et al. 2016; Troscianko et al. 2017a). The effectiveness of camouflage phenotypes generated using CamoEvo can also be validated in field experiments using calibrated printed targets against the natural background (Cuthill et al. 2005; Kjernsmo et al. 2020) providing the background photos used were also calibrated.

## Concluding Remarks

CamoEvo provides a free open‐source resource for running camouflage evolution psychophysics experiments. By using living observers to drive evolution as opposed to camouflage measures, CamoEvo can produce effective camouflage in a short number of generations allowing for potential optima within the phenotypic space to be compared for a variety of treatments. However, this can also serve as a demonstration of the process of adaptation through evolution and the principles of animal camouflage. There are many possible avenues of expansion for the application of CamoEvo including systems specialized for the printing of real‐world targets and the use of custom multidimensional color spaces tailored to specific taxa (similar to how the egg colors are generated). We hope that CamoEvo can be used to aid in the design of future experiments or as a hypothesis generation tool when investigating the interactions between background structure, observer, and variation on camouflage adaptations.

## AUTHOR CONTRIBUTIONS

Initial genetic algorithm approach conceived by JT and then expanded upon and implemented by GRAH. JT wrote the initial animal pattern generation system. All subsequent code for CamoEvo, its genetic algorithm (ImageGA), and egg pattern generation was written by GRAH. Manuscript and user guide first draft written by GRAH, with subsequent edits by JT. Data analyses were performed by GRAH.

## CONFLICT OF INTEREST

The authors declare no conflict of interest.

## DATA ARCHIVING

The dryad doi is https://doi.org/10.5061/dryad.08kprr54d. All data for Box 1 can be found on dryad and our GitHub. Downloads and handbooks for CamoEvo and its genetic algorithm ImageGA can also be found on our GitHub.

2

Associate Editor: Dr. Joseph A Tobias

Handling Editor: Prof. Tracey Chapman

## Supporting information

Supplementary information

Supplementary information

Supplementary information
