# Supplementary material for: CamoEvo: An open access toolbox for artificial camouflage evolution experiments
Source: Evolution. 2022 Mar 30;76(5):870–82. doi: 10.1111/evo.14476 (PMC9314924; doi:10.1111/evo.14476)
Supplement: Supplementary file 1 — Supplementary information [file EVO-76-870-s001.docx]

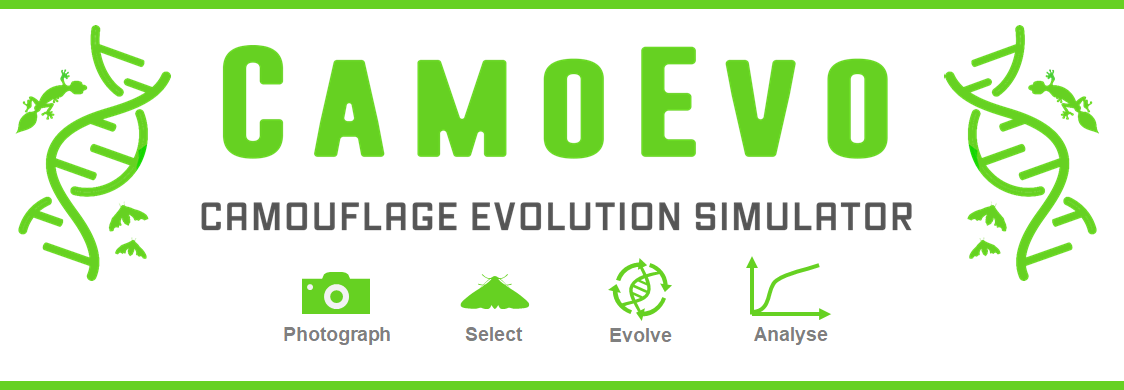


# Contents

[Contents 1](#_Toc90421507)

[Introduction 2](#_Toc90421508)

[What is CamoEvo? 2](#_Toc90421509)

[How to Run a Game 2](#_Toc90421510)

[Installation 2](#_Toc90421511)

[Collecting Images 2](#_Toc90421512)

[Starting a Game 3](#_Toc90421513)

[Playing the game 4](#_Toc90421514)

[Obtaining Data 4](#_Toc90421515)

[How Targets are Generated? 5](#_Toc90421516)

[Animal Patterns 5](#_Toc90421517)

[Egg Patterns 8](#_Toc90421518)

[Shading and Details: 11](#_Toc90421519)

[How Slides are Generated? 12](#_Toc90421520)

[How to run your own Image Analyses 12](#_Toc90421521)

[Additional CamoEvo features 13](#_Toc90421522)

[Phenotype Plotter 13](#_Toc90421523)

[Evolution gif 13](#_Toc90421524)

[Phenotype Maker 13](#_Toc90421525)

[How to Customise CamoEvo 13](#_Toc90421526)

[Changing the GA Settings 13](#_Toc90421527)

[Creating Custom Target Shapes 13](#_Toc90421528)

[File and Code Organisation 16](#_Toc90421529)

[1 CamoEvo: 16](#_Toc90421530)

[2 CamoReq: 16](#_Toc90421531)

[References 17](#_Toc90421532)

# Introduction

Here we describe the features of the camouflage evolution toolbox CamoEvo, how the it functions and how it can be used and modified for research. For examples of teaching practicals using CamoEvo see the Biological Sciences higher biology and Undergraduate practical sheets. For details regarding its genetic algorithm, refer to the ImageGA Guide*.* These files can be found on [GitHub.](https://github.com/GeorgeHancock471)

# What is CamoEvo?

Just as evolution by natural selection provides a mechanism with which organisms adapt to their environment so too does evolution provide a tool for optimising solutions for problems with large parameter spaces. In computer science, genetic algorithms (GAs) are a heuristic global optimisation tool which mimic natural selection to solve complex problems (Goldberg and Holland, 1988). CamoEvo combines the game-like design of common psychophysics experiments, the search mechanisms of modern genetic algorithms and the image analysis features of the MICA toolbox and ImageJ to demonstrate the influence of natural selection on camouflage (Schneider et al., 2012, Bonney et al., 2014, Troscianko et al., 2017, Niu et al., 2018). This program is intended to be used as a research and teaching tool both in the lab and at home, and as a method for designing future camouflage optimisation experiments. CamoEvo outputs key camouflage statistics, graphs, and the stimuli from every round of the game should the user wish to run their own analyses (Troscianko and Stevens, 2015, Troscianko et al., 2017).

# How to Run a Game

## Installation

CamoEvo can be downloaded from either from  [GitHub](https://github.com/GeorgeHancock471?tab=repositories&q=&type=public&language=) or our [Website.](https://www.visual-ecology.com/) When downloading be sure to use the link specified for each operating system (Linux, Mac, and Windows). If you download CamoEvo in this way it should consist of ImageJ bundled with JAVA from NIH as well as CamoEvo, ImageJ and the MICA toolbox (Troscianko and Stevens, 2015).

## Collecting Images

To play CamoEvo you will need to obtain a collection of images which you will evolve your camouflage population on. These images can be taken from anywhere be it a garden, out in the field or online (Figure1). Ideally you should use a number of photos equal to the number of individuals within your population; by default, this is 24. These images should be uploaded to the device which you installed CamoEvo to and placed within a unique folder solely containing these photos. For experiments you’ll want to use photos that are colour calibrated and taken under diffuse lighting, as otherwise variation in colouration between background images may be an artefact of photography as opposed to natural variation and you won’t be able to recreate the targets for field experiments.


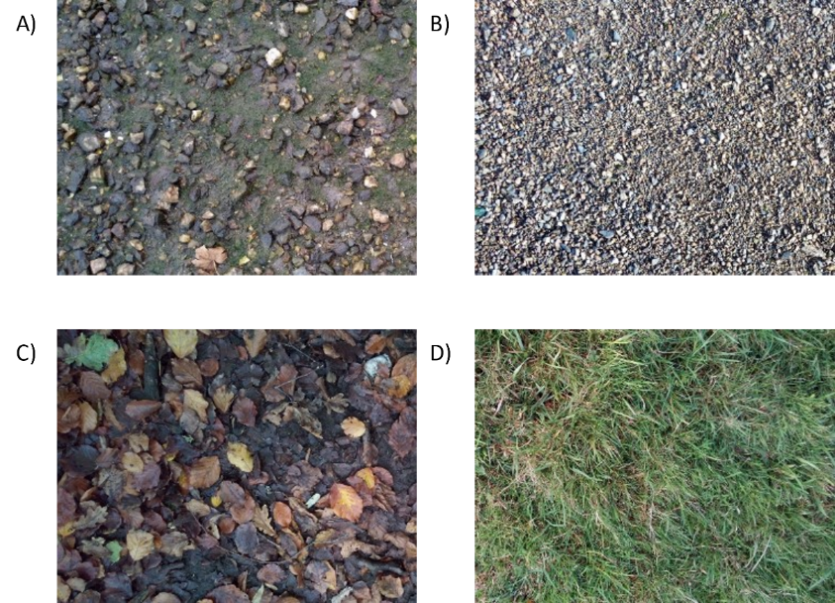


***Figure 1****. Example habitat photos taken from various locations at equal distance: A) estuary gravel, B) estuary pebbles, C) leaf litter and D) wet grass. Photos A) and B) were taken under direct lighting (cloudless day) and so have strong shadows a cue which the target will lack.*

## Starting a Game

Using the UI:

Load up CamoEvo from ImageJ. The CamoEvo plugin should start automatically when launching ImageJ with the version downloaded with CamoEvo pre-packaged. However, should it not, it can be loaded from *plugins/1 CamoEvo/CamoEvo Game*. You can start a new game by clicking new game or you can load an existing game by clicking load game (Figure 2).

When starting a new game, you will need to upload photos by selecting the get photos button and choosing the folder containing the photo gallery you wish to use for your backgrounds. Once you have selected a gallery, you can choose the type of targets you wish to use. You can use targets designed for camouflage experiments (targets), animal shapes (animals) or create your own custom shape using the creator. Different shapes use different pattern generation systems. The targets (triangle, circle, tear, oval) use reaction-diffusion. The animals use the same, but with bilateral symmetry and the egg targets use a unique pattern generation system based on egg maculation theory.

You will then be able to choose, if you wish, from several advanced settings. These include the number of generations (how long the game will be played for), the population size (the number of individuals per generation), group size (the number of individuals shown at a time), time out time (how much time is given to find the targets), colour spaces (the colour range of the populations) and selection pressure (camouflage/signalling) Once you choose the settings you desire, you must give your population a unique name before continuing to play.

We also include two demo populations Camo.Egg and Camo.Toad


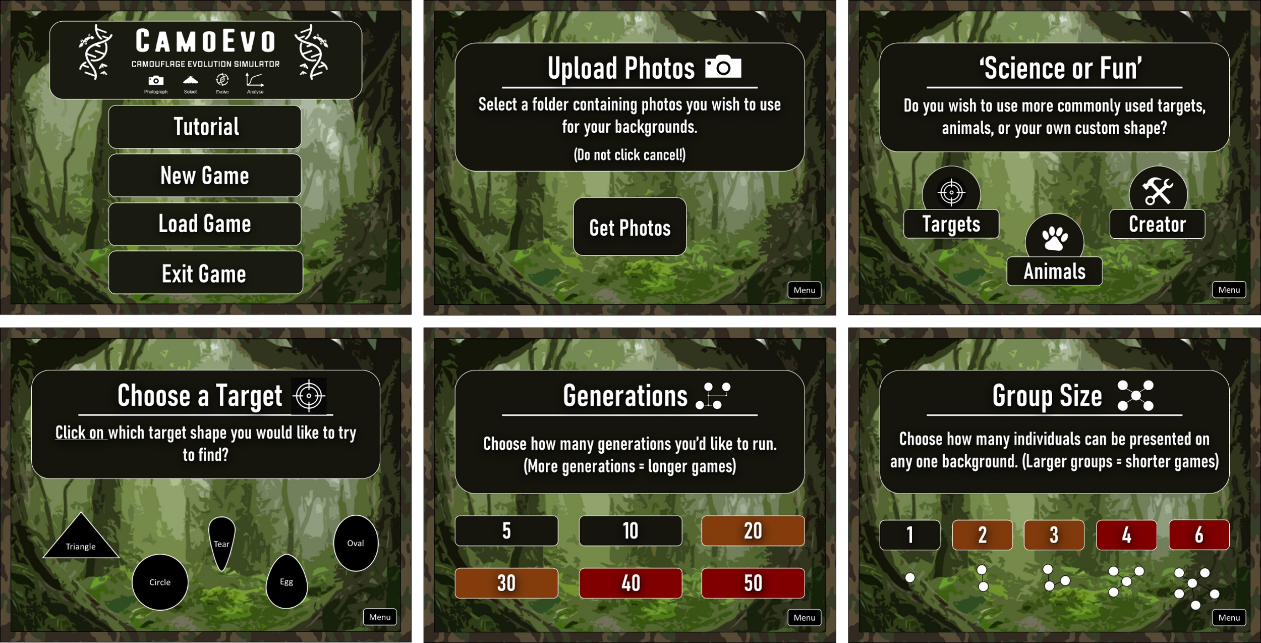


***Figure 2****. Example UI from CamoEvo for setting up the game. (Top Left) CamoEvo main menu, (Top Centre) photo selection, (Top Right) target category. (Bottom Left) target choice, (Bottom Centre) number of generations, (Bottom Right) number of individuals in the population.*

Manually Creating a Game:

For experiments you will likely wish to manually create a game. To do you will need to have a copy of the default population (Population Settings) and game settings (Settings_Game.txt) files. These can be found within the CamoReq folder. All of the settings are labelled within each file. You can also using the game settings file alter additional features such as the target spawn and capture methods or the image cropping method.

You will need to create a folder containing these two files and an additional folder titled /Backgrounds/ with all your background images. Then run *‘/2 CamoReq/GameModule/Camouflage_Evolution.txt’* and select the folder you created.

## Playing the game

Once a population has been setup CamoEvo will automatically ‘evolve’ the first generation. This will typically take 30 to 60 seconds; you will then be given the option to continue to play or exit back to the main menu. The game tasks you with searching for each individual in the population as fast as possible. Between each generation you will be given the option to exit and come back later.

Once you click play you will be asked to move your cursor to the centre of the screen and hold it there. The circle at the centre of the screen will gradually turn white and once completely white you will be presented with a slide featuring one of the backgrounds you uploaded, and a number of targets equal to the group size selected. You will then have to click on all the targets as fast as you can, only moving your cursor from the centre when you spot a target. If you fail to spot the target in time the game will move onto the next screen regardless. Once you have done this for all individuals, the next generation will evolve.

When the final generation is complete you can either run an analysis on the completed generations or click to continue playing. If you click to continue playing the game will run for an additional 5 generations before giving you the option to analyse them again. All camouflage measures are done post game to reduce the load time between each generation.

## Obtaining Data

Once all the generations have been completed you will be presented with the option to run an analysis or to keep playing for 5 more generations. If you click analyse, it will take 1-2 minutes per generation for CamoEvo to go back through each slide of the game and measure the luminance (CIE LAB Δμ luminance), contrast (CIE LAB ΔσL) and colour (CIE LAB Δμ A and B) difference between the target and the local background (McCamy, 1992, Troscianko and Stevens, 2015, Troscianko et al., 2016, Troscianko et al., 2017). Where the local background is a sub sample of the background within a circle of a diameter equal to 2x the diameter of the target. In addition, the level of edge-disruption (CIE LAB GabRatL) of the target against the background is also recorded.


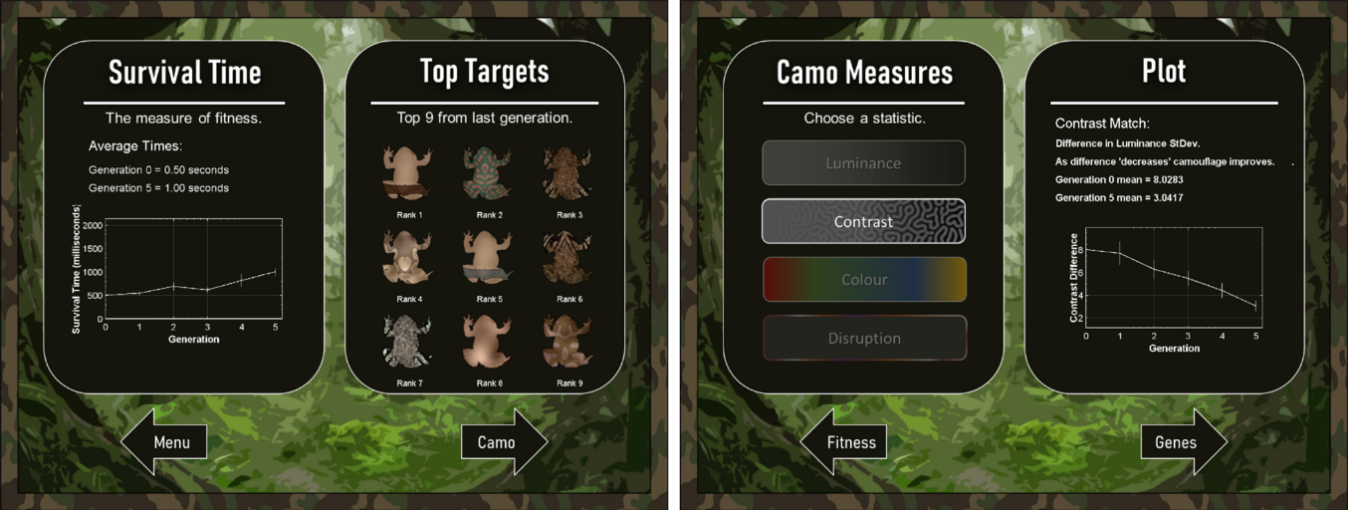


***Figure 3****. Example analysis output from a population of toad targets on boggy grass after 5 generations. (Left) the capture time for each generation and (right) the camouflage statistics with the contrast match for each generation on display.*

Once analysis is complete a results menu with the top 9 individuals from the most recent generation and plots for capture time, luminance match, contrast match, colour match and GABRAT will become available (Figure 3). The plots display the mean and standard error for the population at each generation. These measures can then be extracted as a .txt file for analysis in R or another similar programs. Within the output file there are three types of capture time recordings present. (Capture Time) the time taken for the target to be clicked on, (Reaction Time) the time taken for the cursor to be moved from the centre of the screen, and (Survival Time) which is equal to Reaction Time if $(Reaction time \geq\left( Capture Time - 600ms \right))$, else it is equal to Capture Time. Along with the camouflage recordings the target ID, generation number, background image ID, target X coordinate, target Y coordinate, target rotation and target flip are all recorded and the genome for each individual within the population is also concatenated to the output .txt file. This allows users to run analyses for changes in camouflage phenotype and genotype with generation, the influence of generation on capture time and to account for random factors such as background image and target location. The µL*, µa*, µb* and õL* values for the local background and the targets are also output.

# How Targets are Generated?

## Animal Patterns

Genes

The 39 genes for the animal pattern generation are divided up into the following main sections (lvl1 divisions): pattern genes (14), edge enhancement genes (6), colouration genes (11) and speckling genes (8), see Table 1.

***Table 1****. The pattern generation genes for the reaction-diffusion pattern generation.*

| Gene Name | Function |
| --- | --- |
| ptn_dim_xcp | X coordinate that the pattern is sampled from. |
| ptn_dim_ycp | Y coordinate that the pattern is sampled from. |
| ptn_dim_wdt | The width of the pattern sampled, changes spatial scale. |
| ptn_dim_asr | The aspect ratio of the pattern sampled, stretches (creates stripes). |
| ptn_dim_agl | Rotation of the sampled pattern. |
| ptn_grd_cvr | Percentage of image covered by maculation. |
| ptn_grd_sig | Pattern gradient. |
| ptn_grd_hgt | The height of the gradient. |
| ptn_grd_agl | The angle of the gradient. |
| eem_int_lvl | Edge enhancement intensity level. |
| eem_int_rto | Edge enhancement intensity ratio of light and dark. |
| eem_sig_lvl | Edge enhancement sigma (level of blurring). |
| eem_sig_rto | Edge enhancement sigma ratio of light and dark. |
| eem_exp_lvl | Edge enhancement expansion (size). |
| eem_exp_rto | Edge enhancement expansion ratio of light and dark. |
| ptn_bil_ctr | The position of bilateral asymmetry. |
| ptn_bil_sft | The intensity of y transform. |
| ptn_bil_frq | The frequency of y transform. |
| col_mac_lmv | Colour maculation luminance channel (L*). |
| col_mac_rgv | Colour maculation green-red channel (a*). |
| col_mac_byv | Colour maculation blue-yellow channel (b*). |
| col_top_lmv | Colour top luminance channel (L*). |
| col_top_rgv | Colour top green-red channel (a*). |
| col_top_byv | Colour top blue-yellow channel (b*). |
| col_bot_lmv | Colour bot luminance channel (L*). |
| col_bot_rgv | Colour bot green-red channel (a*). |
| col_bot_byv | Colour bot blue-yellow channel (b*). |
| col_grd_sig | Colour gradient sigma between top and bottom. |
| col_grd_hgt | Height of the colour gradient. |
| spk_one_lvl | Intensity of speckling 1. |
| spk_one_rto | Ratio of light to dark speckling 1. |
| spk_one_sig | Gaussian sigma of speckling 1. |
| spk_one_ycp | Y coordinate speckle sheet 1. |
| spk_two_lvl | Intensity of speckling 2. |
| spk_two_rto | Ratio of light to dark speckling 2. |
| spk_two_sig | Gaussian sigma of speckling 2. |
| spk_two_ycp | Y coordinate speckle sheet 2. |

Maculation

Animal maculation is generated using Gray-Scott reaction diffusion (Allen et al., 2010, Kondo and Miura, 2010). Given that most of the reaction-diffusion space (F and K values) result in blank black or grey spaces, our system uses a non-linear selection of the space. We did this by manually selecting (subjectively) the bounds of interesting patterns, then creating a model (4th degree polynomial in this case) to work out K-min and k-max from F. From these values a 2D pattern plot is generated and used to provide the gamut of reaction-diffusion patterns used by CamoEvo (Figure 4) . New plots can be generated using "\CamoReq\Reaction_Diffusion_Pattern_Generator_gradient_smlap_adaptivek.java".


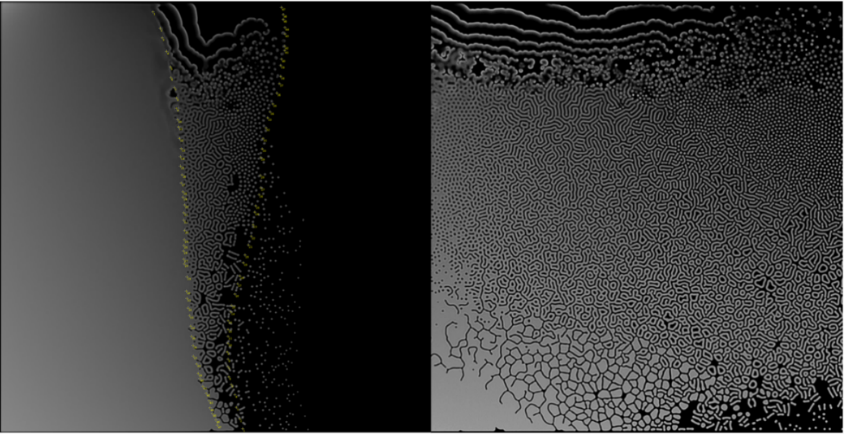


***Figure 4****. Left: the generated Gray-Scott reaction-diffusion space and the polynomial selected points shown in yellow. Right: the output 2D pattern space for the polynomial K and linear F.*

It is from this reaction-diffusion plot that a region is sampled using the genome and then rotated and scaled to produce additional maculation patterns. For example, increasing the aspect ratio converts spots into stripes (Figure 5). Using a pattern sheet, in this way, saves time as the lengthy reaction-diffusion pattern generation does not need to run for each individual and ensures perfect heritability of maculation. Maculation can also be set to be mirrored bilaterally and all the targets with animal shapes are set to do this by default. For examples of animal maculation see Figure 6.


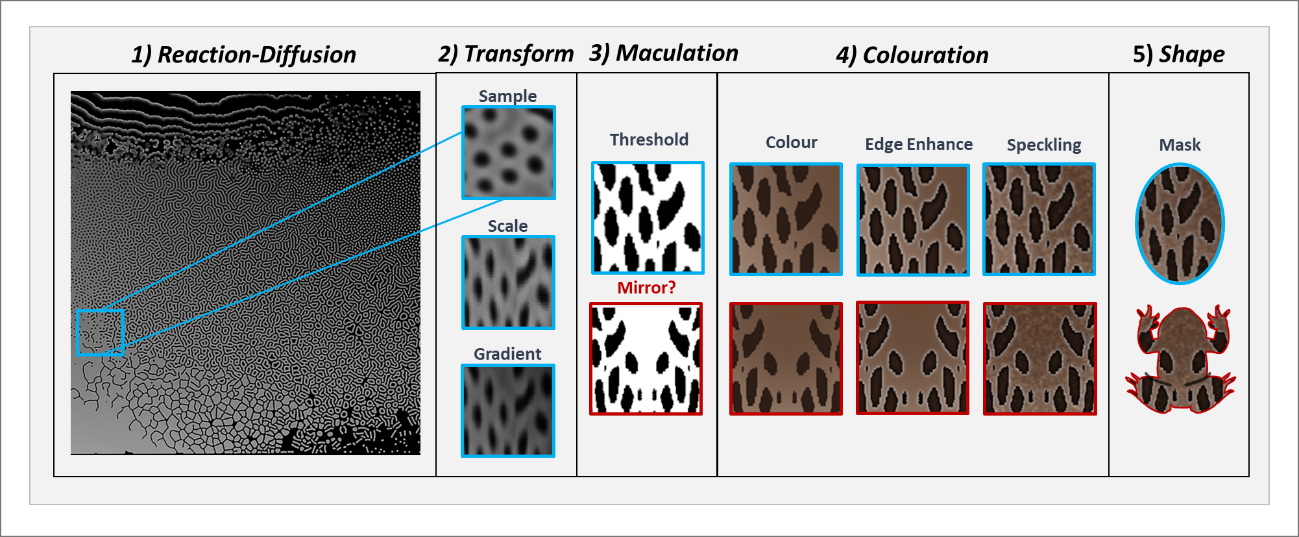


***Figure 5****. Schematic of the animal pattern generation system used by CamoEvo. A section of the reaction-diffusion gamut is selected, scaled and shaded with a gradient before being thresholded. Depending on what target shape is selected the pattern is mirrored bilaterally. Colouration is then applied to both the background and the pattern in addition to edge enhancement and speckling. All these features are regulated by different genes.*

*
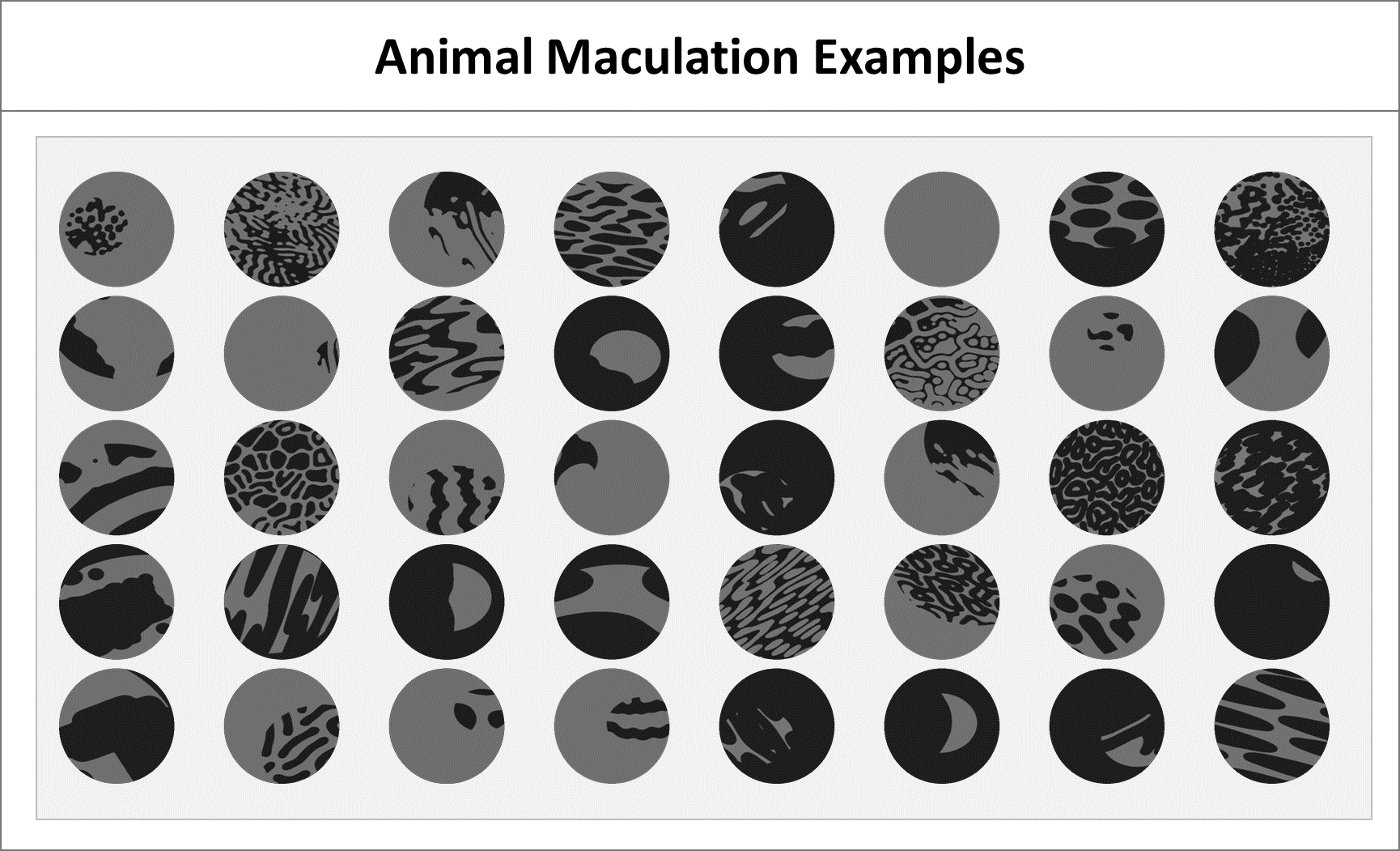
*

***Figure 6****. Examples of random over dispersed maculation, showing the background in light grey and the maculation in dark grey. All genes not relating to maculation were switched off.*

Colouration

Animal colouration is generated using the CIELAb colour space (McCamy, 1992). The L*, a* and b*values can be adjusted in order to impose biological limitations or to speed up the optimisation of colour by limiting the population to the colouration of the background (Figure 7).


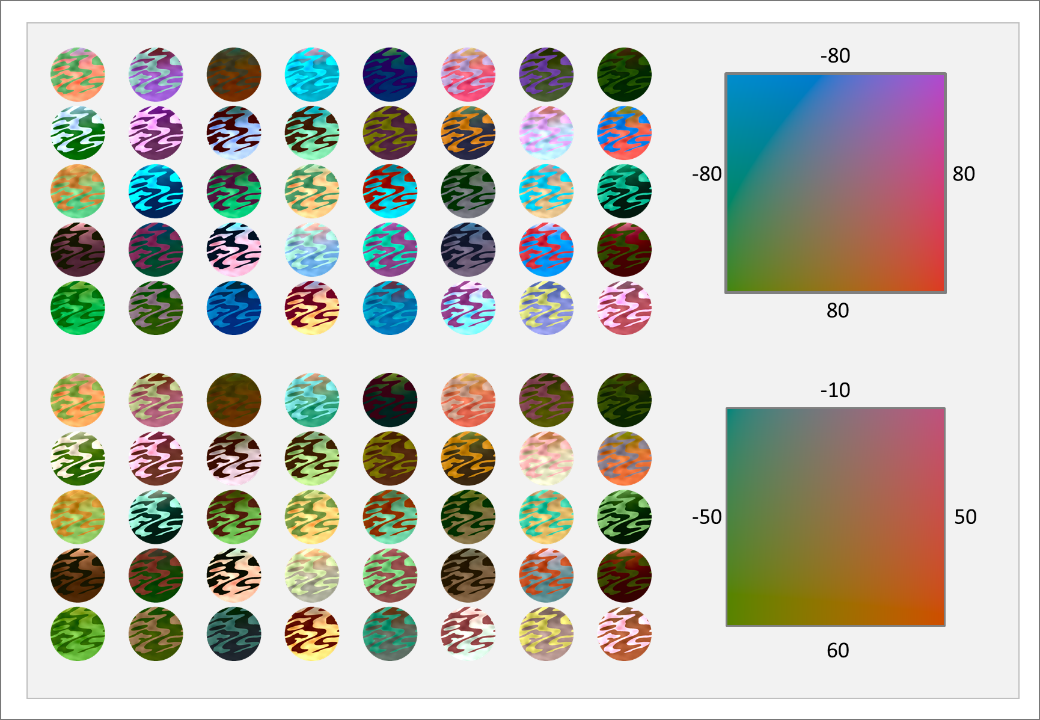


***Figure 7****. Colouration generated using two different colour spaces. Above shows a broader colour space with a* and b* ranges of -80 to 80. Below shows the same population but with the colour space narrowed to an a* range of -50 to 50 and -10 to 60.*

There are three different regions of colouration the maculation, the top of the background and the bottom of the background. The colour of the background is created using a gradient of the top and bottom colours and there are two genes that regulate the height and sigma (smoothness) of the gradient (Figure 8).


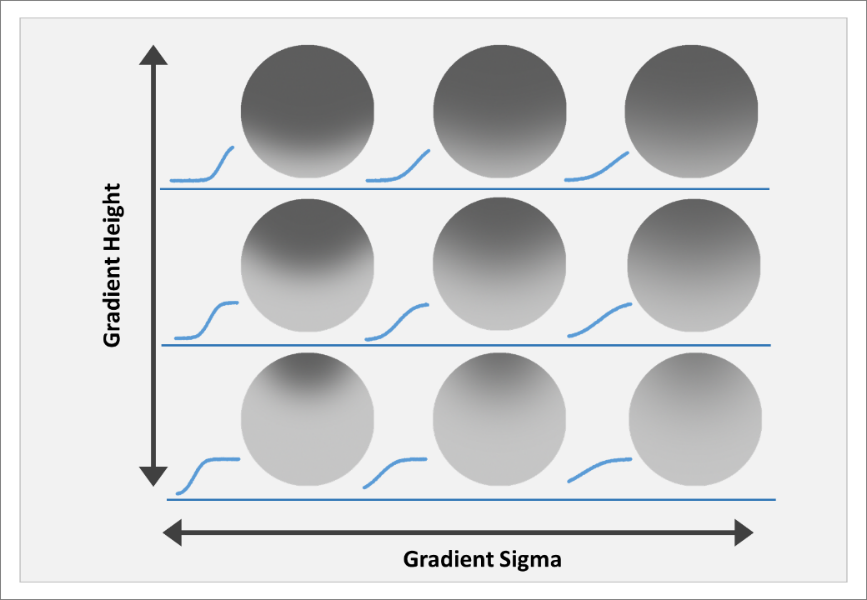


***Figure 8****. The shading genes for background colouration. Using change in luminance as an example (shading does include a* and b* normally). The luminance change from the top most to bottom most point on the image is shown to the left of each circular target.*

Edge Enhancement

Edge enhancement is created by increasing the difference in luminance at the border (dark gets darker and light gets lighter) between the maculation and then adjusting the sharpness of the slope and breadth of the peak between the increased and original contrast (Figure 9) (Egan et al., 2016, Sharman and Lovell, 2019). For each of these characteristics (intensity, sigma and expansion) there is also a ratio gene which alters the value between the light and dark region allowing for asymmetry in the shape of the wave. *Note: the average luminance for the maculation and the background is conserved, by re-adjusting the mean after edge enhancement is applied.*


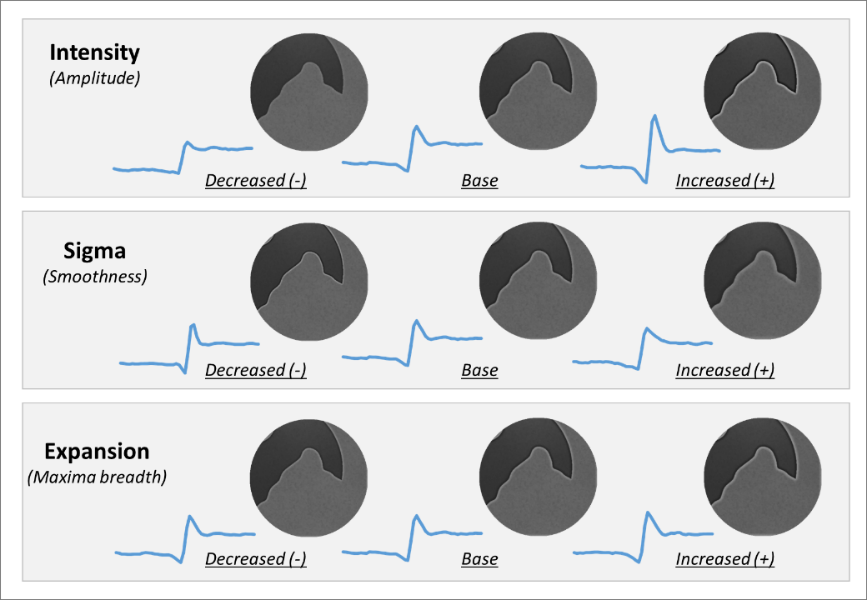


***Figure 9****. The edge enhancement genes for animal pattern generation. The middle for each row shows the same base pattern. The left shows the pattern with the corresponding value decreased and the right shows the value increased. The graph to the bottom left of each image shows the luminance transect taken perpendicular to the edge of the maculation and the background. Increasing the intensity increases the contrast. Lower sigma values produce sharper peaks and higher values lead to smoother peaks. Increased expansion makes the peak flatter.*

Speckling

In addition to maculation, speckling patterns are generated for the intensity channel using random noise and Gaussian blurring to create patterns of varying intensity and spatial scale (Figure 10_. This is repeated twice to allow for noise at different spatial frequencies. Like edge enhancement the intensity of the light and dark maculation is regulated by ratio genes. The random noise for the speckling is sourced from two different noise sheets to insure perfect heritability. The Y-coordinate can also be shifted to allow the position of the speckling to be adjusted slightly. *Note: the average luminance for the maculation and the background is conserved, by re-adjusting the mean after speckling is applied.*


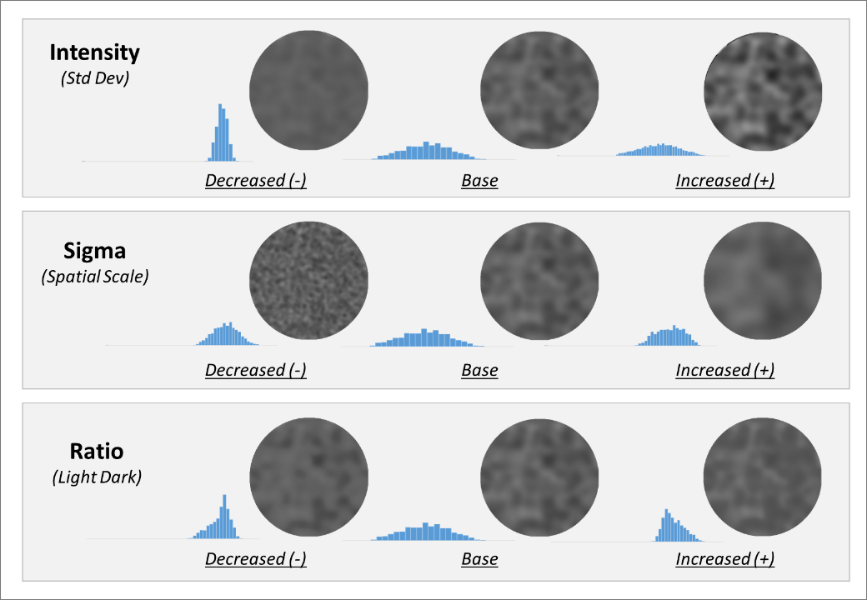


***Figure 10****. The speckling genes for animal pattern generation. The middle for each row shows the same base pattern. The left shows the pattern with the corresponding value decreased and the right shows the value increased. The graph to the bottom left of each image shows the histogram for luminance values of the shape. Increasing the intensity increases the standard deviation of luminance. Increasing the sigma value lowers the spatial frequency. Lower ratios decrease the deviation of values greater than the original mean and higher ratios decrease the deviation of values darker then the mean.*

## Egg Patterns

Genes

The 31 genes for the egg pattern generation are divided up into the following main sections: pores (2), patterns (20) and colour (9).

Egg pore patterns are generated with the same speckling method as the reaction-diffusion generation system though at a reduced range.

***Table 2****. The pattern generation genes for the Gaussian egg pattern generation.*

| gene name | Function |
| --- | --- |
| por_int_lvl | Pore intensity. |
| por_sig_lvl | Pore size. |
| pat_noi_int | Speckling of background. |
| pat_noi_sig | Speckling blur. |
| pat_noi_thr | Threshold for speckles. |
| pat_mc1_xcd | Maculation 1 x coordinate. |
| pat_mc1_ycd | Maculation 1 y coordinate. |
| pat_mc1_thr | Maculation 1 threshold. |
| pat_mc1_sig | Maculation 1 sigma. |
| pat_mc1_med | Maculation 1 median filter (creates splattered pattern). |
| pat_mc1_noi | Maculation 1 additional noise. |
| pat_grd_blr | Gradient sigma. |
| pat_grd_hgt | Gradient height. |
| pat_grd_lng | Gradient length. |
| pat_grd_int | Gradient intensity. |
| pat_mc2_xcd | Maculation 2 x coordinate. |
| pat_mc2_ycd | Maculation 2 y coordinate. |
| pat_mc2_sig | Maculation 2 sigma. |
| pat_mc2_thr | Maculation 2 threshold. |
| pat_mc2_rnw | Maculation 2 random walk duration (creates squiggle patterns). |
| pat_mac_noi | Internal maculation noise. |
| pat_mac_smg | External maculation smudging. |
| col_bkg_dep | Amount of pigment added to background. |
| col_bkg_pir | Ratio of pigment added to background. |
| col_pt1_dep | Amount of pigment added to maculation 1. |
| col_pt1_pir | Ratio of pigment added to maculation 1. |
| col_pt1_rep | Percentage of maculation 1 deposition in copied pattern. |
| col_pt2_dep | Amount of pigment added to maculation 2. |
| col_pt2_pir | Ratio of pigment added to maculation 2. |
| col_pt2_rep | Percentage of maculation 2 deposition in copied pattern. |
| col_exp_lvl | Egg exposure. |

Maculation

Unlike the other targets, eggs use their own distinct pattern generation system based on egg maculation and pigment colour theory (Pike, 2015, Pike, 2019). Instead of using reaction-diffusion to generate the maculation, thresholding Gaussian noise and Gaussian blurred images are used to create the speckled and spotted patterns of eggshells. Like the reaction-diffusion a pattern sheet is used to reduce generation time and to improve heritability (Figure 11). This pattern sheet consists of a 1-dimensional change in Gaussian noise where Gaussian noise increases along the y axis.

A gradient is applied to each pattern prior to thresholding to allow for the belted and pole concentrated patterns of eggs seen in birds. Spiral patterns are generated by applying random walk and copying the pattern to different locations (Pike, 2015). Each egg can have up to 4 maculation patterns allowing for different patterns such as spots, speckles and spirals to exist concurrently. For simplicity patterns 3 and 4 are copies of patterns 1 and 2, respectively. These copies are shifted on the X-axis such that they are identical in terms of pattern generation, without being the exact same pattern. Large blotches are created by thresholding, blurring and re-thresholding patterns 1 and 3. Spirals are created by applying random walk to patterns 2 and 4. Smeared patterns are generated by blurring the outside of the maculation. Unlike animal maculation thresholding is not binary, this allows the level of deposition to slope from the maximum and towards that of the background. Currently random walk pattern is not perfectly heritable. For examples see Figure 12.


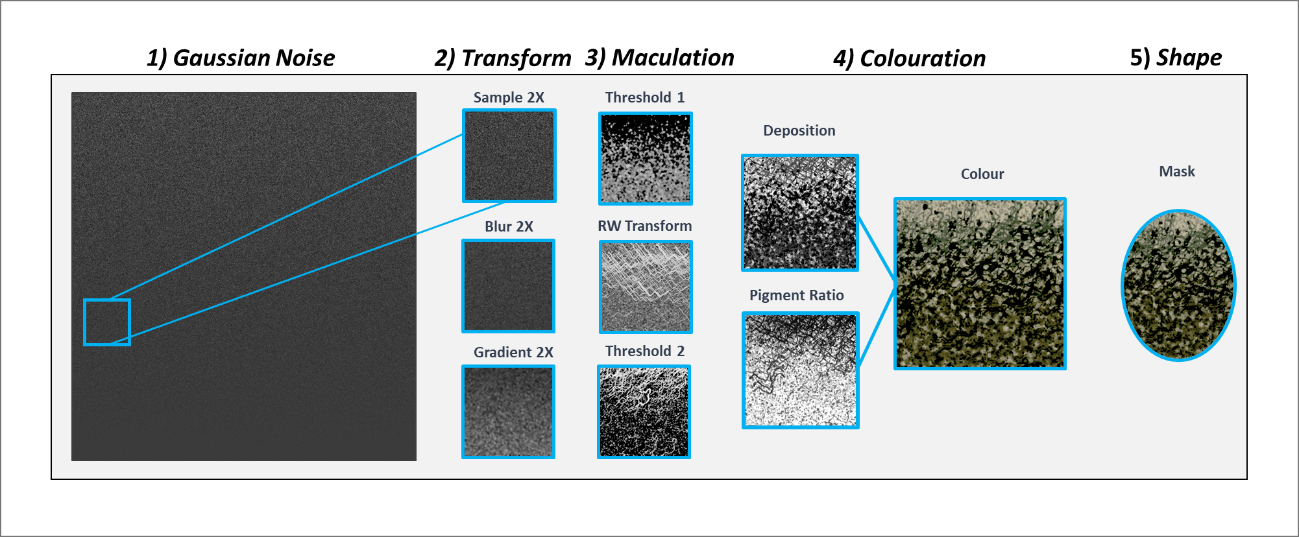


***Figure 11****. Schematic of the egg pattern generation system used by CamoEvo. A section of the Gaussian noise gamut is selected, blurred and shaded with a gradient before being thresholded. Colouration is then applied to both the background and the patterns.*


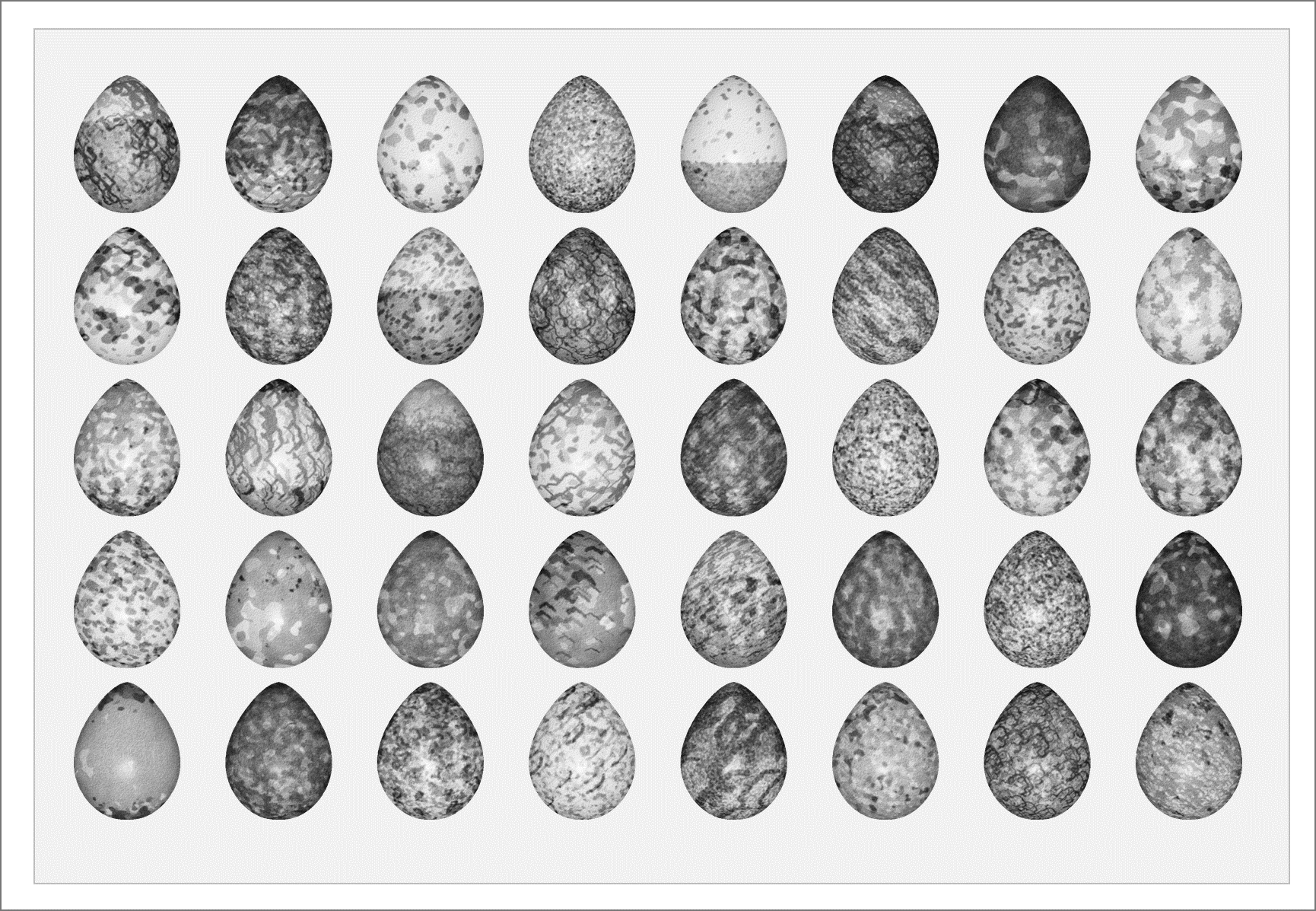


***Figure 12****. . Examples of random over dispersed egg maculation, showing the background in light grey and the maculation in dark grey. All genes not relating to maculation were switched off. As maculation is additive and thresholding is not binary, intensity varies where maculation 1 and maculation 2 collide and with the gradient curves of the noise map the maculation’s are created from.*

Coluration

Egg background and maculation colour uses a CIELAB colour space and is controlled using genes for pigment ratio and pigment deposition. Pigment type determines the ratio of the red-yellow pigment protoporphyrin and the green-blue pigment biliverdin. While pigment deposition determines the amount of pigment added.(Hanley et al., 2015, Wisocki et al., 2020) (Figure 13). Increasing the amount of protoporphyrin increases the a* and b* values, though extreme concentrations decreases the b* value. Increasing the amount of biliverdin decreases the a* and b* values. Both pigments decrease the L* value but protoporphyrin does so by a greater amount. The CIELAB colour is calculated by multiplying the pigment L*, a* and b* values by their ratio and deposition. Maculation colour is calculated by adding the deposition to that of the background (maculation are always darker) and by averaging the pigment ratio weighted by deposition. The maculation copies (patterns 3 and 4) have their pigment ratio taken from their copied maculation but have their own deposition. The luminance of maculation patterns and is calculated by subtracting the luminance from that of the background. Like animal patterns the colour range of the eggs can be adjusted. For more examples see Figure 14.


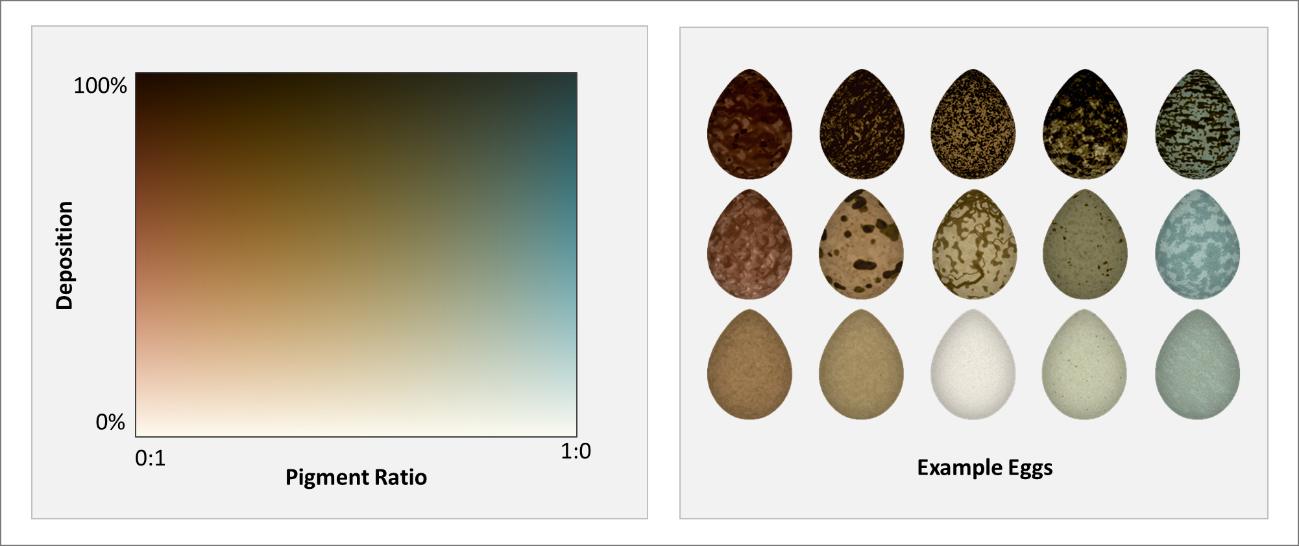


***Figure 13****. Egg Colouration Gamut.* *Deposition and Pigment Ratio operate in combination to change the CIE LAB colour of the eggs. Deposition determines how much pigment is added and so makes the eggs darker. Ratio determines how much of each pigment is added. Lower ratio = more protoporphyrin and higher ratio = more biliverdin. The left shows the full colour gamut and the right shows example eggs of varying average pigment ratio and average depositon.*

*
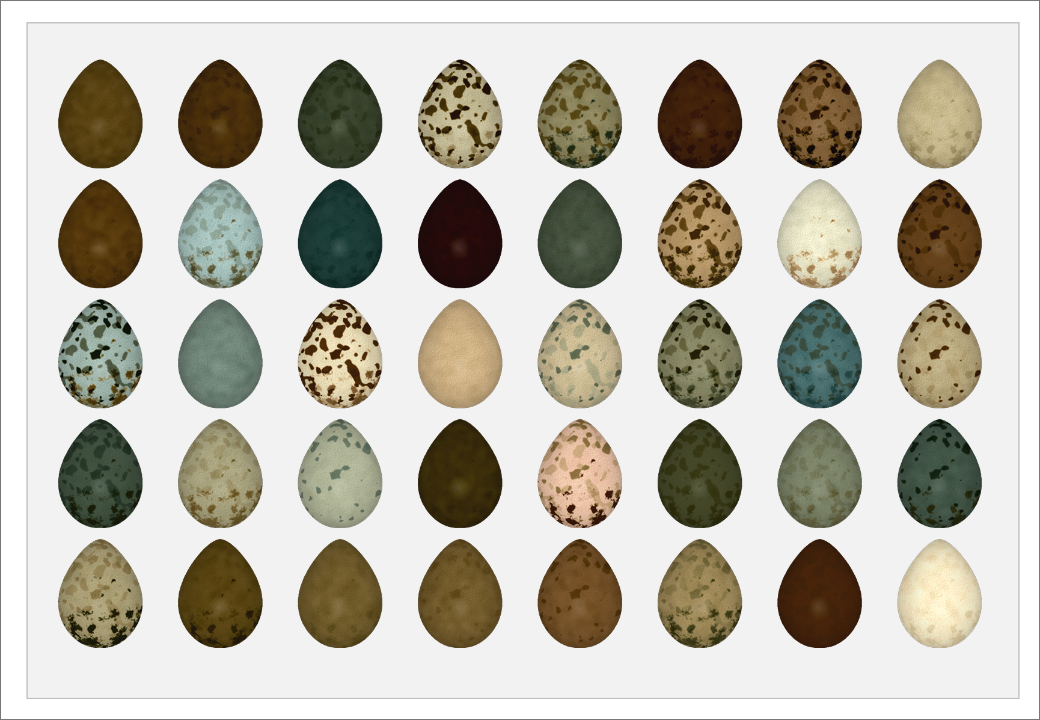
*

***Figure 14****. Example egg colouration for 40 overdispersed randomly generated eggs. All genes barring those that regulate colour were locked such that only deposition and pigment ratio varied for all 4 maculations and the background of the egg.*

## Shading and Details:

For both animal and egg patterns additional shading and details can be added. *Note this is not a stand in for real world lighting effects.* These can be used to force targets to have pattern or shape cues that they have to evolve around. The contrast of both the shading and details can be adjusted within the target settings make it easier to make multiple iterations of different targets. All the shader maps and details for the default targets were drawn by hand using the mask source image as a reference.

Shading

Shading works by using an RGB image with mean 127. Regions that are above 127 will appear brighter and regions that are below 127 appear darker. The map created is divided by 127 and then multiplied by the target pattern (See Figure 15).

Details

Details work by using an alpha channel to combine the target image with a custom image. This can be used to add eyes, display patterns or specular reflectance like effects (See Figure 15).

*
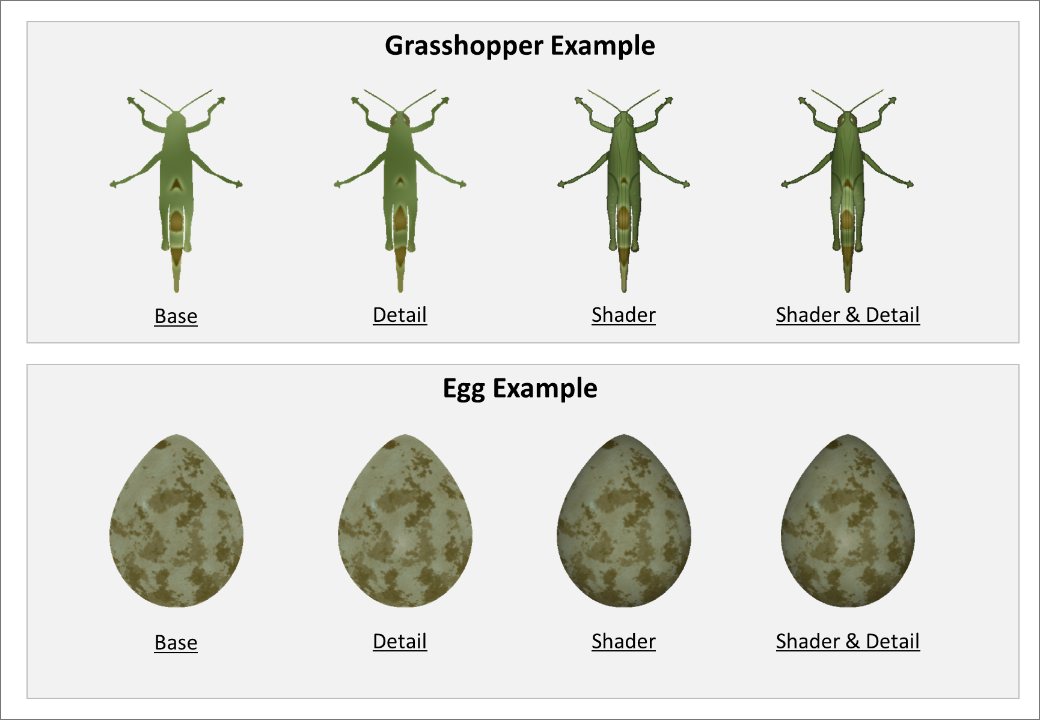
*

***Figure 15****. Examples of the detail and shader system using gasshoppers and eggs. For each the base unaltered target is shown along with the same target with just the detail, just the shader and both the detail and shader. The grasshopper detail is the presence of eyes that are shifted towards brown and the egg instead has a small specular patch. The shader for the grasshopper gives it depth and clear segmentation, while the egg shader just provides depth.*

## How Slides are Generated?

Each CamoEvo slide is generated using one of the background images and a number of targets equal to the group size selected. The sequence with which the targets and the backgrounds are selected is randomly generated. If the number of backgrounds provided is lower than the number of slides necessary to display all the targets, additional random sequences of backgrounds will be generated and concatenated until the sequence is equal to the number of slides required. This means that depending on the number of backgrounds provided, not all backgrounds will be displayed the same number of times.

When adding targets to each background the target is assigned a random x and y coordinate and rotation. The range of x and y coordinates are restricted such that the target cannot spawn within the target’s diameter of the centre. This prevents targets from being immediately located due to being at the observer’s point of focus (Talas et al., 2020). For each target, a random angle between -180^0^ and +180^0^ is assigned (. This angle range can be adjusted for each target type by editing the "/1 CamoEvo/Targets/Target_Settings.txt" file.

If the group size is greater than one, multiple targets will be added to each background. Each time a target coordinate is assigned, the distance between the target and existing targets is calculated and the coordinates adjusted to prevent targets from spawning too close to each other, though this will fail if the targets are too big compared to the background.

In addition before and after the target is applied a custom script can be run. This can be used to alter the image colour, randomise the target pose or add an occlusion layer to name a few examples.

# How to run your own Image Analyses

If you wish to run your own custom image analyses, the slides presented by CamoEvo are all saved as .txt files. You can use the regenerate_stimuli function to recreate the slides and the regions of interest (target and local background) selections.

# Additional CamoEvo features

## Phenotype Plotter

Allows you to select a CamoEvo population and generate a plot showing the phenotype (appearance) of individuals from each generation. The interface allows you to adjust the space between rows and columns, the number of individuals shown per generation, the range of generations used, the interval between generations, the ordering of individuals and the labels. The ordering can either be: ranked, individuals are shown in order of fitness; inverse, individuals are shown in reverse order of fitness; category, individuals are shown in the order they are in the chromosome file and age, oldest to youngest. *Note: the plot image is quite large and may need to be down-scaled before saving.*

## Evolution gif

Allows you to create a gif version of the phenotype plot showing each generation as slices as opposed to columns.

## Phenotype Maker

Allows you to test the influence of genes and colour space on phenotype and to create custom starting genomes for experiments.

# How to Customise CamoEvo

## Changing the GA Settings

Using CamoEvo’s interface you can adjust the number of generations and the population size used by its genetic algorithm. However, if you wish to change other aspects of the algorithm e.g., mutation rate or crossover probability you will need to change the default settings that CamoEvo uses with ImageGA.

To prevent other users of ImageGA from interfering with or changing CamoEvo, we provide a separate settings storage .txt file for CamoEvo so that CamoEvo’s settings can be adjusted independently. We also provide an interface macro ‘/1 CamoEvo / Edit Algorithm.txt’ specifically for changing the settings used by CamoEvo that are not altered by the advanced settings (number of generations and population size). The genetic algorithm settings can also be manually adjusted by changing the “AlgorithmSettings.txt” file within the population folder.

By default, CamoEvo uses the following:

Deletion Pool = **66%**

Breeding Pool = **33%**

Mutation Pool = **0%**

Point Mutation Prob = **0.001**

Lvl1 Mutation Prob = **0.0125**

Lvl2 Mutation Prob = **0.0125**

Link Mutation Prob = **0.3333**

Gene Displace Prob = **0.1**

Rescue Number = **3**

Crossover Type = **random**

Crossover Probability = **1.0000**

Recombination = **incomplete**

Mating System = **random**

Breeding Pool Assignment = **ranked**

## Creating Custom Target Shapes

As individuals using CamoEvo are likely to want to tailor the size and shape of targets to their own experiments we provide an interface for creating custom targets. Users can either import existing targets they’ve made for other experiments or create new targets. To make a target type you need an image of the shape you want. This image needs to be a binary black and white 400x400px image where the target shape is drawn in white. The target should fit entirely within a 400px diameter circle. We provide a template circle for the purposes of creating targets. Alternatively, you can draw a shape using the creator itself.

Once you have picked a shape you can then choose which pattern generation system the target will use (animal maculation, animal bilateral maculation or egg maculation) and the size of the target. To make target scaling easier the target will be displayed against a sample background from the background image gallery chosen.

If you do not want to use the creator, you can manually create new targets by copying one of the rows in the "/1 CamoEvo/Targets/Target_Settings.txt" file and adjusting the values. You will also need to create a folder containing the target shape containing the target image titled “target.png”. The columns are organised as follows:

*Note: the target size, min and max angle is copied to the psychophysics setting by the CamoEvo UI. If you want to alter the settings after instead change the Settings_Game*

| *ID:* | *The unique ID for the target type distinguishing it from the others.* |
| --- | --- |
| *Shape:* | *The name of the folder from which the target shape is taken.* |
| *Size* | *The size in pixels of the target.* |
| *Pattern:* | *The type of pattern generation used Targets / Eggs* |
| *Centre:* | *The X coordinate of the sagittal plane for bilateral symmetry.* |
| *minAngle:* | *Minimum angle of rotation.* |
| *maxAngle:* | *Maximum angle of rotation.* |
| *Shading α:* | *The alpha for the applied shading (if used).* |
| *Details α:* | *The alpha for the detail (if used).* |
| *Bilateral?:* | *Yes or No to bilateral symmetry* |

# Psychophysics Game

## Requirements

To set up a game you will need a folder containing the following:

- *Backgrounds/ :* A folder containing background images only.
- *Targets/ :* A folder containing target images with alpha as 0.
- *Training/ :* A folder containing dummy images shown at the beginning.
- *Settings_Population.txt :* A .txt file containing the game settings.

## Game Settings

These are the setting that influence the setup of the psychophysics game.

*Note, if setting up for Camouflage Evolution experiments you need to initialise the game before adjusting these settings. I.e. the first population (generation_0) needs to be created first. Else the min and max angle*

| *TargetSize:* | *The size in px of the target, (future includes % and cm).* |
| --- | --- |
| *NumPerSlide:* | *Number of targets shown per slide (max = 6, min = 1).* |
| *Angles:* | *The tab delimited min and max angle of the target.* |
| *TimeOutTime:* | *The time limit for each slide.* |
| *SpawnMethod:* | *Where targets can spawn on the screen (see below).* |
| *CaptureMethod:* | *How capture is determined (see below).* |
| *TransitionMethod:* | *The method use to change slide, mouse to centre, click anywhere or wait.* |
| *TrainingNumber:* | *The number of training targets used (see Settings Population).* |
| *Repeats:* | *The number of repeats for each trarget (not currently used for evolution)* |
| *ClickLeeWay:* | *The % increase around the target where a click is considered a hit.* |
| *CropWidth:* | *The X dimension for cropping (can’t be bigger then source image).* |
| *CropHeight:* | *The Y dimension for cropping (can’t be bigger then source image).* |
| *CropMode:* | *Where the image is cropped (image centre or random)* |
| *FailTrigger:* | *What is considered a fail (see below).* |
| *TargetMod:* | *Modifications applied to the target (see below).* |
| *ImageMod:* | *Modifications applied to the whole image (see below).* |

Spawn Method

Targets can either spawn randomly on the screen or be selected to choose locations that are closer of further in distance in either the L, a*, b* or normalised combined channels. Future versions will include grid spawning and clustered spawning where multiple individuals will spawn together rather than apart from one another.

*Note: targets will not spawn off the screen or overlapping the centre of the screen (fixation point).*

Capture Method

Determines how successful capture of the target is determined.

- *click:* clicking on the target is considered capture.
- *response:* moving the mouse is considered capture.
- *left&right:* if the target is on the left hand side of the screen left-click else right-click
- *rank:* same as click but asks the user to click in order of conspicuousness for multiple targets.

Fail Method

Determines what is considered a fail. Fails result in an immediate time out.

- *none:* Only a time out is considered a fail.
- *miss:* clicking anywhere other than the target is considered a fail.
- *react:* moving the mouse creates a time window of 600ms once that elapses the slide ends.
- *miss/react:* both missing and reacting are considered fails.

Target and Image Modifiers

Allows the user to run custom scripts that apply to the target or the whole image. An example of a target modifier and an image modifier is given in figure 6. Potential uses for this include applying randomised postures, filters mimicking different visual systems or objects that can occlude the targets.


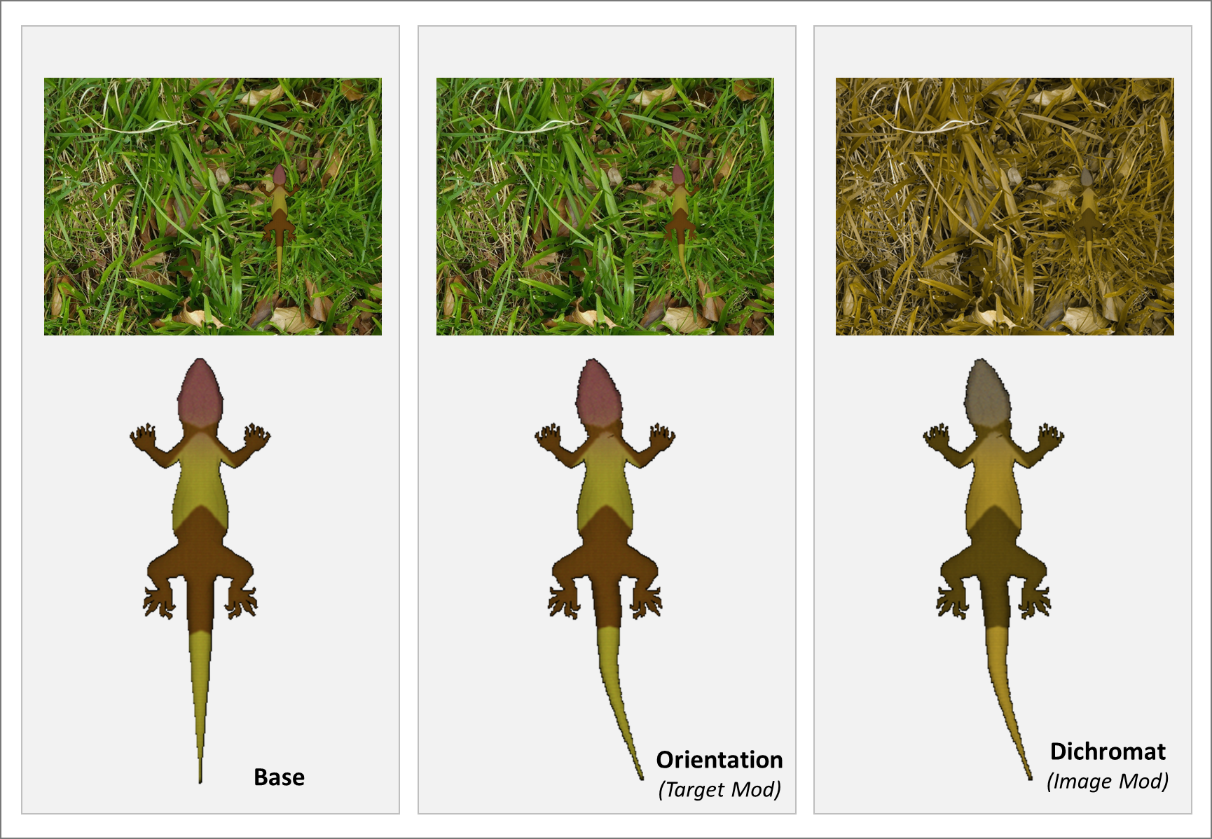


***Figure 16****. Examples of target and background modifiers. An orientation modifier randomises the rotation of the head and the spline of the tail. While a dichromatic filter is applied to the whole image including the target.*

## Population Settings

These are the settings that are specific to running the Camouflage Evolution Game, though there is some overlap between the Game Settings and Genetic Algorithm as some of the settings are copied over to from here to the Algorithm_Settings.txt and Settings_Game.txt files.

| *Target:* | *The name of the target type and the relevant settings* |
| --- | --- |
| *Generation:* | *The number of generations of evolution.* |
| *PopSize:* | *The number of individuals in the population for each generation.* |
| *NumSlide:* | *The number of individuals per slide.* |
| *TimeOutTime:* | *The time limit for each slide.* |
| *StartSpace:* | *The range of colours of the starting population (see below).* |
| *ColourSpace:* | *The range of colours the population can explore (see below).* |
| *FitnessValue:* | *What determines fitness Camouflage or Signalling (inverse value).* |
| *MeasureMethod:* | *The value used for fitness (survival time, response time, capture time or rank).* |
| *Transition:* | *The slide transition method (see Game Setttings).* |
| *DemoImage:* | *How demo images are created (see below).* |
| *DemoCount:* | *How many demo images are used (see Game Setttings).* |

Colour Spaces

The user can adjust the colour space ranges of the starting population and of the entire evolution run. Note the colour space should never be narrower than the starting space.

For animal pattern generation the values correspond to:

min.L* max.L* min.a* max.a* min.b* max.b*

For egg pattern generation the values correspond to:

min.D max.D min.Pr max.Pr blank blank

DemoImage

Generates dummy images that appear at the start of the game. These can either be randomly generated or resurrected individuals from the previous generations.

- *None:* no dummy images
- *First:* only shows dummy images for the first generation
- *Every:* shows randomly generated targets at the start of every generation
- *Resurrect:* shows randomly generated targets for the first generation then dead targets from the previous generation for the following generations

# File and Code Organisation

For the purposes of making CamoEvo easier to edit, the code is organised as follows:

## 1 CamoEvo:

Folder containing all of the plugins intended to be used at the outset by CamoEvo. This includes the CamoEvo starting interface, the phenotype and evolution plotters and the GA settings interface:

- Interface/: *Contains the UI and a .pptx file with all the original slides used to make the UI.*
- Population/: *Contains the folders for all the populations created.*
- Targets/: *Contains all the Target types including the custom generated ones.*
- CamoEvo_Game: *Runs CamoEvo.*
  - Contains the UI for setting up a game and loading previous games.
  - Contains a built-in tutorial.
- Edit Algorithm: *Allows the user to edit the settings of ImageGA that aren’t changeable using the built in UI (Mutation, Mating Systems and Crossover).*
- Get_Phenotype_Plot: *Customisable plot of ranked targets over multiple generation.*
  - Customizable X and Y interval
  - Customizable column spacing
  - Targets are ordered by fitness, top left = fittest, bottom right = least fit
- Get_Evolution_gif: *Creates an animation showing the evolution of the entire population.*
  - *Same customizations as the plots*
- Phenotype_Maker: *Allows you to test the effects of gene changes and colour space, import genomes and create custom genomes.*
- Regenerate_Stimuli: *Recreates the stimuli and Roi’s for a select generation.*
- Regenerate_Targets: *Recreates all the target images using the genome stored in the data_output file.*

*.*

## 2 CamoReq:

Contains all the requisit files for running CamoEvo and a number of the .txt files used to store information. Most importantly among these are:

- Colour Maps/: *Contains the code and corresponding maps for the CIELAB egg and animal spaces.*
  - colour_map_animal = the animal camouflage CIE LAB space
  - colour_map_egg = the egg CIE LAB space
- ExamplePatterns/: *Demo Patterns used by UI*
- GameModule/: *Contains the code for the psychophysics game, screen scaling and the evolution game.*
  - Camouflage Evolution = run evolution on target folder.
  - Psychophysics Game = runs game with backgrounds and folders.
  - Get Screen Scales = gets the dimensions for the computer screen.
- Mod_Stimuli/: *Background Stimuli Modifiers*
- Mod_Targets/: *Target Modifiers*
- Patterns/: *Contains the reaction-diffusion and gaussian pattern sheets*
  - eggEdgeNoise.png = noise durinng egg maculation expansion
  - eggPatterns.jpg = egg patterns
  - pattern1.jpg = animal patterns
  - speckle1.tif & speckle2.tif = noise maps for animal speckling
- Target_Gen/: *Contains the phenotype makers and gene templates.*
  - Egg_Gene_Template: The gene template used for egg patterns.
  - Gene_Template: *The gene template for the animal patterns.*
  - Generate_Eggs: *The code for converting the egg pattern genes to their phenotype.*
    - *Output images are not saved using this script*
    - *As batch mode is true, output images aren’t visible*
  - Generate_Targets: *The code for converting the animal pattern genes to their phenotype.*
    - *Output images are not saved using this script*
    - *As batch mode is true, output images aren’t visible*
- Colour_Range_Modifier: *Adjust the colour range of the starting population by altering gene values.*
- Reaction_Diffusion_Pattern_Generator: *Creates reaction-diffusion patterns.*
- Reaction_Diffusion_Pattern_Generator_gradient_smlap_adaptivek: *Creates reaction-diffusion pattern sheets.*

# References

ALLEN, W. L., CUTHILL, I. C., SCOTT-SAMUEL, N. E. & BADDELEY, R. 2010. Why the leopard got its spots: relating pattern development to ecology in felids. *Proceedings of the Royal Society B: Biological Sciences,* 278**,** 1373-1380.

BONNEY, R., SHIRK, J. L., PHILLIPS, T. B., WIGGINS, A., BALLARD, H. L., MILLER-RUSHING, A. J. & PARRISH, J. K. 2014. Next Steps for Citizen Science. *Science,* 343**,** 1436.

EGAN, J., SHARMAN, R. J., SCOTT-BROWN, K. C. & LOVELL, P. G. 2016. Edge enhancement improves disruptive camouflage by emphasising false edges and creating pictorial relief. *Scientific Reports,* 6**,** 38274.

GOLDBERG, D. E. & HOLLAND, J. H. 1988. Genetic Algorithms and Machine Learning. *Machine Learning,* 3**,** 95-99.

HANLEY, D., GRIM, T., CASSEY, P. & HAUBER, M. E. 2015. Not so colourful after all: eggshell pigments constrain avian eggshell colour space. *Biol Lett,* 11**,** 20150087.

KONDO, S. & MIURA, T. 2010. Reaction-Diffusion Model as a Framework for Understanding Biological Pattern Formation. *Science,* 329**,** 1616.

MCCAMY, C. S. 1992. Correlated color temperature as an explicit function of chromaticity coordinates. *Color Research & Application,* 17**,** 142-144.

NIU, Y., SUN, H. & STEVENS, M. 2018. Plant Camouflage: Ecology, Evolution, and Implications. *Trends in Ecology & Evolution,* 33**,** 608-618.

PIKE, T. W. 2015. Modelling Eggshell Maculation. *Avian Biology Research,* 8**,** 237-243.

PIKE, T. W. 2019. Quantifying the maculation of avian eggs using eggshell geometry. *Ibis,* 161**,** 686-693.

SCHNEIDER, C. A., RASBAND, W. S. & ELICEIRI, K. W. 2012. NIH Image to ImageJ: 25 years of image analysis. *Nature Methods,* 9**,** 671-675.

SHARMAN, R. J. & LOVELL, P. G. 2019. Edge-Enhanced Disruptive Camouflage Impairs Shape Discrimination. *i-Perception,* 10**,** 2041669519877435-2041669519877435.

TALAS, L., FENNELL, J. G., KJERNSMO, K., CUTHILL, I. C., SCOTT-SAMUEL, N. E. & BADDELEY, R. J. 2020. CamoGAN: Evolving optimum camouflage with Generative Adversarial Networks. *Methods in Ecology and Evolution,* 11**,** 240-247.

TROSCIANKO, J., SKELHORN, J. & STEVENS, M. 2017. Quantifying camouflage: how to predict detectability from appearance. *BMC Evolutionary Biology,* 17**,** 7.

TROSCIANKO, J. & STEVENS, M. 2015. Image Calibration and Analysis Toolbox – a free software suite for objectively measuring reflectance, colour and pattern. *Methods in Ecology and Evolution,* 6.

TROSCIANKO, J., WILSON-AGGARWAL, J., STEVENS, M. & SPOTTISWOODE, C. N. 2016. Camouflage predicts survival in ground-nesting birds. *Scientific Reports,* 6**,** 19966.

WISOCKI, P. A., KENNELLY, P., ROJAS RIVERA, I., CASSEY, P., BURKEY, M. L. & HANLEY, D. 2020. The global distribution of avian eggshell colours suggest a thermoregulatory benefit of darker pigmentation. *Nature Ecology & Evolution,* 4**,** 148-155.
