# Supplementary material for: CamoEvo: An open access toolbox for artificial camouflage evolution experiments
Source: Evolution. 2022 Mar 30;76(5):870–82. doi: 10.1111/evo.14476 (PMC9314924; doi:10.1111/evo.14476)
Supplement: Supplementary file 2 — Supplementary information [file EVO-76-870-s003.docx]

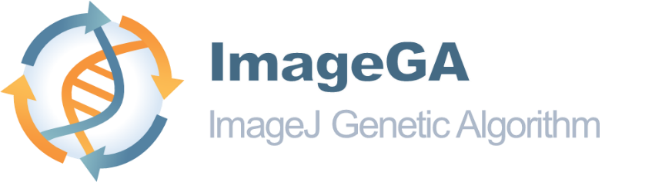


# Contents

[Contents 1](#_Toc62562736)

[Introduction 2](#_Toc62562737)

[What is ImageGA 2](#_Toc62562738)

[How does ImageGA work? 2](#_Toc62562739)

[Running ImageGA for the first time. 2](#_Toc62562740)

[Downloading and Installing ImageGA 3](#_Toc62562741)

[Create a Directory folder 3](#_Toc62562742)

[Run ImageGA demo 3](#_Toc62562743)

[ImageGA Settings 3](#_Toc62562744)

[Biological Sex 3](#_Toc62562745)

[Starting Populations 4](#_Toc62562746)

[Pool Units 4](#_Toc62562747)

[Deletion Pool 4](#_Toc62562748)

[Breeding Pool 4](#_Toc62562749)

[Number of Generations 4](#_Toc62562750)

[Mutation Rate 4](#_Toc62562751)

[Mutation Distribution 4](#_Toc62562752)

[Mutation Strength 4](#_Toc62562753)

[Crossover Type 4](#_Toc62562754)

[Crossover Probability 5](#_Toc62562755)

[Recombination Completeness 5](#_Toc62562756)

[Mating System 5](#_Toc62562757)

[Inversion Mutation Probability 6](#_Toc62562758)

[Duplication Mutation Probability 6](#_Toc62562759)

[Scramble Mutation Probability 6](#_Toc62562760)

[Targeted Duplication Mutation Probability 6](#_Toc62562761)

[Crowd Operators 6](#_Toc62562762)

[ImageGA Outputs 7](#_Toc62562763)

[Individual IDs 7](#_Toc62562764)

[Parents 7](#_Toc62562765)

[ImageGA Fitness Calculators 7](#_Toc62562766)

[Travelling Salesperson 7](#_Toc62562767)

[Background Match 8](#_Toc62562768)

[Creating your own Gene Templates 9](#_Toc62562769)

[Targeted Duplication and Gene Units 9](#_Toc62562770)

[Creating Your Own Fitness Calculators 10](#_Toc62562771)

[File and Code Organisation 10](#_Toc62562772)

[/Fitness Calculators/: 10](#_Toc62562773)

[/RequisitFunctions/: 10](#_Toc62562774)

[ImageGA Demo: 11](#_Toc62562775)

[Recommended Settings for Psychophysics Experiments 11](#_Toc62562776)

[References 11](#_Toc62562777)

# Introduction

Here we describe the features of the genetic algorithm plugin ImageGA in addition to two worked examples of its use i) travelling salesperson problem and ii) camouflage evolution experiment. These examples are designed to showcase the functionality of ImageGA and how it may be used by researchers wishing to conduct their own evolution experiments. The guide as it is published here was designed for ImageGA v2.0, documentations detailing future versions can be found  [GitHub](https://github.com/GeorgeHancock471?tab=repositories&q=&type=public&language=) or our [Website.](https://www.visual-ecology.com/) ImageGA was created for the camouflage evolution simulator CamoEvo. For the documentation explaining how to use CamoEvo refer to the [CamoEvo_Supplement.](https://github.com/GeorgeHancock471/User_Guides/archive/main.zip)

# What is ImageGA

ImageGA is a genetic algorithm (GA) Toolbox for ImageJ designed to aid in survival experiments with evolving ImageJ generated patterns. The plugin is designed to be largely customisable, with built in options for population size, genotype, mutation rates, crossover type and mating system.

ImageGA comes with a built-in template for performing two versions of the Travelling Salesperson problem: a ring-shaped map of 16 cities, and a map of the USA with 48 of the 50 state capitals (excludes Hawaii and Alaska). For each of these problems, Gaussian noise can be added to the data to simulate noise which will naturally occur in other optimisation problems such as the psychophysics experiments ImageGA was designed for.

ImageGA is automatically included with CamoEvo and is required for CamoEvo to run correctly.

# How does ImageGA work?

ImageGA, like most genetic algorithms, relies on classical evolutionary theory in order to produce populations which increase in fitness with each generation (Goldberg and Holland, 1988). Similar to evolution by natural selection, only the surviving (fittest) members of a population get to breed producing offspring that are recombinants of their parents and have their own unique mutations. Over time the fitness of the population is expected to increase as the population fixates on an optimal phenotype.

# Running ImageGA for the first time.

## Downloading and Installing ImageGA

ImageGA can be obtained either by downloading CamoEvo or independently from either on [GitHub](https://github.com/GeorgeHancock471?tab=repositories&q=&type=public&language=) or our [Website.](https://www.visual-ecology.com/) When downloading be sure to use the link specified for each operating system. If you download ImageGA separately you will need to have ImageJ bundled with JAVA from NIH downloaded already. Not FIJI.

## Create a Directory folder

Before you start running ImageGA you will want to create a new empty directory which you will want to save your generation files to. E.g., /ImageGA Test 1/

This will be used as the Working Directory for ImageGA, for storing all of the Generation, Survival and Phenotype Data.

## Run ImageGA demo

Open ImageJ, go to your plugins folder / ImageGA / and run ImageGA demo.

- Select the Working Directory you created.
- Select ‘Salesperson_Ring_16’ as your Template.
- Choose ‘Create Custom’, then set the population size to 24 and the population type to random.
- Keep the Algorithm Settings Part 1 and Part 2 as default.
- Select ‘Salesperson_ Ring_16’ as your fitness calculator.
- ImageGA should then start to produce 10 generations worth of Survival Data for the Travelling Salesperson problem in addition to creating a folder with the fittest route from each generation.
- Within the directory you should find .txt files for each generation, txt files with the fitness value for each generation (total travel distance) and a folder containing maps of the fittest salesperson routes for each generation. In addition, there will be a .csv file named Fitness_Summary containing the fitness values and IDs for all generations.

# ImageGA Settings

All settings for the ImageGA plugin can be altered within the ImageGA interface, this panel will alter the default settings used by the algorithm. In this section you will find descriptions regarding each of the ImageGA operators, how they work and what they do.

## Biological Sex

Within Genetic Algorithms sex/mating type is a commonly used feature for maintaining genetic diversity within large populations (Rejeb and AbuElhaij, 2000). If you choose to enable biological sex the population will be split into two mating types. Males will only be able to mate with females and females will only be able to mate with males. Additionally, when ranking by fitness, males and females are ranked separately.

## Population Size

Determines how many individuals in a population.

## Number of Generations

Determines how many generations the population will evolve for not including generation 0 which is the starting population.

## Starting Populations

When creating starting populations ImageGA presents you with the option to either create a custom population or use the existing. If you choose to ‘Use Existing’ the gene values and population size of the genotype template will be used. If you instead choose to create custom, you can create a population within intervals of 2. You can then choose whether to use random values, uniformly distributed values, pre-set values, or random mutants from the pre-set values.

## Pool Units

Determines the unit for deleting and breeding individuals within the population can either be integers or fraction. The adjusting the units used makes it easier to tailor the algorithm to populations of specific sizes while percentages are better for handling systems where the algorithm needs to work for populations of multiple sizes.

## Deletion Pool

Determines how many individuals get deleted within the next generation.

## Breeding Pool

Determines the number of individuals that get to breed in the next generation. If the number is fewer then the number deleted, then multiple mating can occur.

## Mutate Pool

Determines the number of survivors who undergo clonal mutation, starting from the worst to best.

## Mutations

The rate of point, lvl1, lvl2 and linked mutations can be set.

Point mutations effect one gene.

Lvl 1 effect all genes that share the 1^st^ label

Lvl 2 effect all genes that share the 2^nd^ label

Linked allow point mutations to influence both genes that share the same 1^st^ and 3^rd^ label.

*Unlike others this is the probability of a point mutation effecting both so it’s probability is also determined by the probability of a point mutation.*

These rates can be adjusted separately for the gametic (before crossover), offspring (post crossover) and clonal.

## Protective Operators

These can be used to preserve the fittest individuals in the population.

You can adjust the number that get cloned, starting from the fittest.

You can provide a lifeline for the fittest in the previous generation, so that it can’t be deleted in the generation immediately after the one it was the fittest in.

Adaptive Operator (Displacement Mutation)

When enabled, shifts offspring away from their parents in both directions, if they are similar to one another.

Adaptive Operator (Scale with Rank)

When enabled, the lower the rank of the parent the higher the mutation probability of its offspring.

Adaptive Operator (Scale with Variance)

When enabled, the lower the variance the lower the mutation probability but mutations are more extreme. Works best with displacement active.

Adaptive Operator (Scale with Fitness)

When enabled, increasing fitness lowers mutation rate and decreasing fitness increases it.

## Crossover Type

Determines the pattern of recombination of genes between parents.

*Random:* all genes have 50/50 chance of coming from either parent.

*One-Point:* one random section of the genome from each parent are crossed over.

*Two-Point:* two random sections of the genome from each parent are crossed over.

Multi-point: random walk of the genome from each parent are crossed over.

## Crossover Probability

Determines the probability that the offspring of two individuals are produced with crossover. Setting this probability to -1 makes it so that all individuals are produced asexually. If crossover does not occur, then the resulting offspring are copies of each parent + any mutations added.

## Recombination Completeness

Determines whether recombination for each gene is perfect or imperfect. If you choose perfect recombination when a decimal gene is copied it is copied in its entirety. If you choose imperfect recombination then each gene will be a value between that of the two parents but weighted towards one of them following the formula. Imperfect recombination of genes can aid with phenotypic exploration by creating offspring with values between existing optima, a benefit usually unique to binary encoding systems as opposed to decimal.

## Breeding Pool

Determines how individuals are assigned to the breeding population. Either ranked selection or binary tournament.

## Mating System

Determines the order of mating within the breeding population. Note Poly, Disassortative, Assortative and Adaptive are all experimental mating systems and so aren’t recommended on release.

*Random:* each individual within the breeding pool is randomly paired with an unpaired individual.

*Ranked:* the individuals within the breeding pool mate in the order of fitness with individuals pairing, starting top to bottom, with the individual of adjacent rank.

*Inverse:* the individuals within the breeding pool mate in the inverse order of fitness with individuals pairing, starting top to bottom, with the individual of opposite rank.

*Disassortative:* starting with the fittest individual the sum Euclidean distance for each gene is calculated between that individual and the remainder of the breeding pool $\sum_{i=1}^{n} d(p_{i}, q_{i})$ (n=number of genes, p = the gene from individual 1 and q = the gene from individual 2). That individual is then paired with the individual which had the greatest difference in genotype until all the breeding pool have been paired.

*Assortative:* starting with the fittest individual the sum Euclidean distance for each gene is calculated between that individual and the remainder of the breeding pool $\sum_{i=1}^{n} d(p_{i}, q_{i})$ (n=number of genes, p = the gene from individual 1 and q = the gene from individual 2). That individual is then paired with the individual which had the lowest difference in genotype until all the breeding pool have been paired.

# ImageGA Outputs

For each generation ImageGA outputs a .txt file containing the genome of every individual within the population for that generation. Each genome is marked by a unique ID signifying the individual.

## Individual IDs

Each individual generated within a population is given a unique ID by ImageGA e.g., Gen23_Mut1_ID18. ‘Gen’ indicates the generation the individual was created in, ‘mut’ indicates the number of times the individual was in the non-breeding population and ‘ID’ indicates the order in which it was created within the generation it was first made in. Regardless of the ID within the gene template, the ID is automatically replaced with an ID in the correct format.

If you are using crowd operators, individuals created through immigration will possess an X next to their ID number, e.g., Gen12_Mut0_IDX1.

## Parents

For each individual in the population, the parents that produced it are recorded with the denotation Par_[ID]. If the individual was created in the first generation it has no parent, ‘none’. If the individual was created clonally then the parent ID will be the same as the individual’s ID.

If you are using crowd operators, immigrant individuals will have their parents labelled as Par_X, unless you are using the mutantMate or randomMate system, in which case only one of the parents will be labelled ParX to indicate that the individual.

| ID | Penzance | StIves | Parent1 | Parent2 |
| --- | --- | --- | --- | --- |
| Gen1_Mut0_ID15 | 0.900208136 | 0.442986907 | Par_Gen0_Mut0_ID10 | Par_Gen0_Mut0_ID28 |
| Gen2_Mut0_ID3 | 0.926931126 | 0.574447599 | Par_Gen1_Mut0_ID19 | Par_Gen1_Mut0_ID0 |
| Gen0_Mut0_ID8 | 0.246032931 | 0.499525392 | none | none |

# ImageGA Fitness Calculators

Fitness calculators also known as fitness functions are the mechanism with which the genotype template is converted to a phenotype and then measured for its fitness. ImageGA features three built in fitness calculators, each with their own gene template. These can be found in the Fitness Calculator folder located within the ImageGA plugin.

## Travelling Salesperson

The travelling Salesperson Problem or TSP is a commonly used genetic algorithm optimisation tool where a list of cities, towns or coordinates are presented, and the algorithm is tasked with finding the shortest route for visiting all the locations. In both the travelling salesperson examples given each gene corresponds with one of the cities on the map provided. The value of the gene represents the order with which the site is visited, with lower values being visited first. The fitness calculator then measures the distance travelled from one site to the next in the sequence of lowest to highest decimal value. The negative sum distance travelled for each route is then outputted as the fitness value, such that the greater the distance the lower the fitness values. We provide a shorter (16 city) and longer (48) city demo for the purposes of testing algorithm settings.

***Salesperson Ring 16***

In this example a salesperson is attempting to visit 16 cities in a ring site (Figure 1). Each gene in the ‘Salesperson_Ring_16.txt’ corresponds with one of the cities and is labelled with the city’s name. The optimal route is shown on the map in grey. Routes generated by the corresponding fitness calculator are shown in red. For each generation, the optimal route is outputted as a picture. If you use the ‘Salesperson_Ring_16_(Noise)’ calculator, Gaussian noise is applied to the fitness value for each individual in each generation. You can adjust the level of Gaussian noise by editing the value in ‘ImageGA/Fitness Calculators/Salesperson/Noise.txt’, the random Gaussian noise is multiplied by the number within the .txt file.


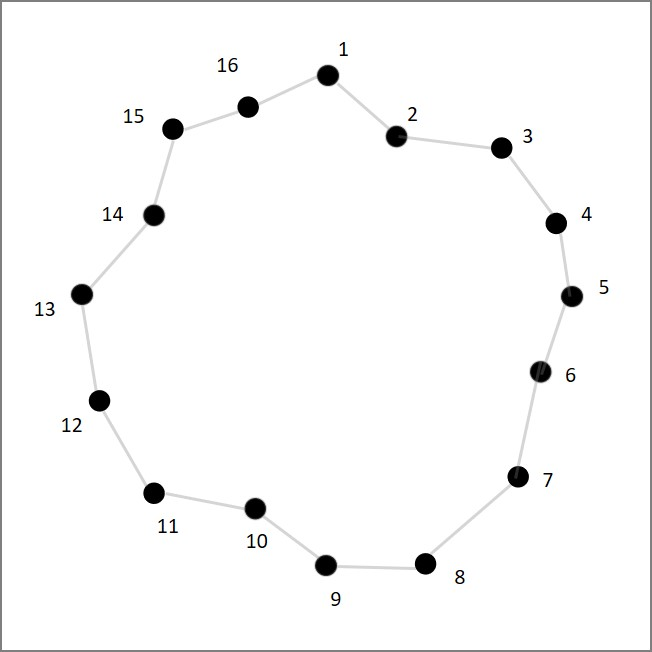


***Figure 1:***  *Ring Map, circular map where the optimal route is a ring shown in grey. Each point represents a location the salesperson needs to visit.*

***Salesperson USA 48***

In this example a salesperson is attempting to visit 48 of the US capitals (Figure 2). Each gene in the ‘Salesperson_USA_48.txt’ corresponds with one of the capital cities and is labelled with the cities name. The optimal route is shown on the map in grey. Routes generated by the corresponding fitness calculator are shown in red. For each generation, the optimal route is shown as a picture. If you use the ‘Salesperson_USA_48_(Noise)’ calculator, Gaussian noise is applied to the fitness value for each individual in each generation. You can adjust the level of Gaussian noise by editing the value in ‘ImageGA/Fitness Calculators/Salesperson/Noise.txt’, the random Gaussian noise is multiplied by the number within the .txt file.


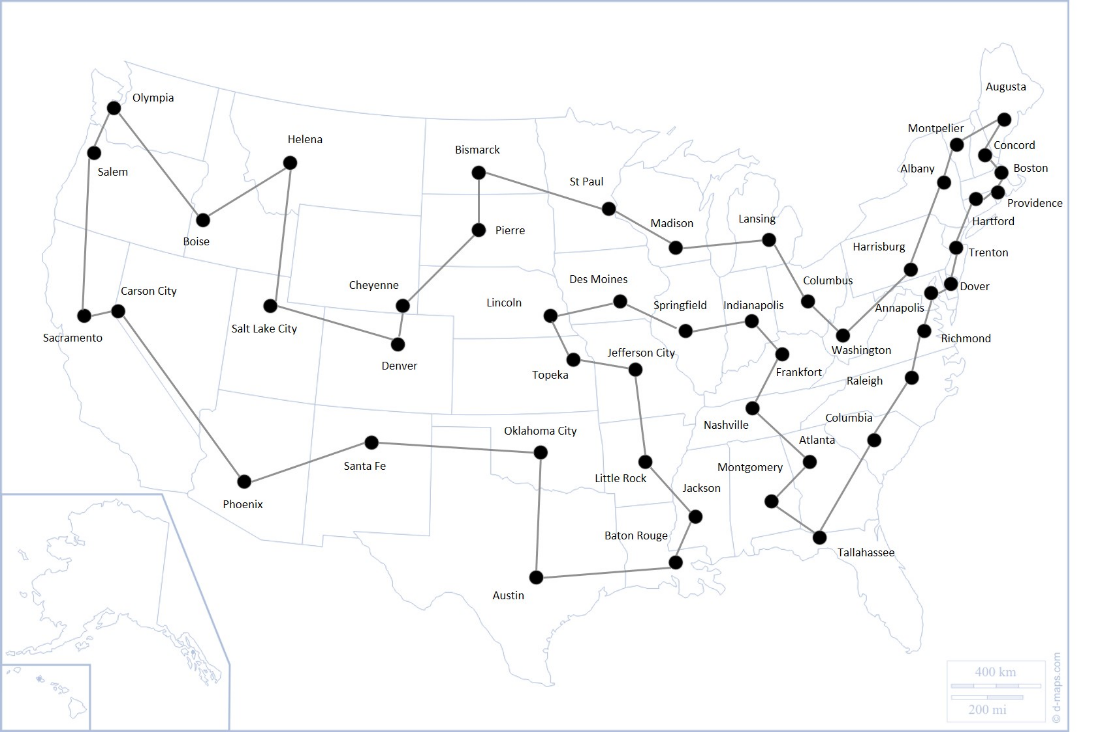


***Figure 2:***  *USA Map of state capitals where the optimal route is a loop shown in grey. Each point represents a capital the salesperson needs to visit.*

# Creating your own Gene Templates

To create a population template, you will need a .csv or .txt file containing a row of ‘headings’. The first heading must always be ‘ID’ this is the number tag which is assigned to each individual within the population (Each individual will be given a Unique ID). This ID consists of the following: **Gen**, the generation it was created in, **Mut** how many times it has been in the mutation pool and **ID** the unique number assigned to its generation. Additional tags include **C** and **R**, C marks individuals that were cloned in the protective system and R marks individuals that were rescued.

Then the following headings are the names given to all of your genes. Currently genes have to have a three point “_” delimited label. Future versions will make this system more flexible.

| ID | Parent1 | Parent2 | Sex | G_N_1 | G_N_2 | G_N_3 | G_N_4 |
| --- | --- | --- | --- | --- | --- | --- | --- |

If you so choose the population template can be filled with pre-existing values for all of the individuals within the population **Note that all Genes must be of a decimal value between 0-1 and there must be enough individuals to breed.**

| ID | Parent1 | Parent2 | Sex | G_N_1 | G_N_2 | G_N_3 | G_N_4 |
| --- | --- | --- | --- | --- | --- | --- | --- |
| Gen0_Mut0_ID0 | - | - | m | 0.630631 | 0.347511 | 0.300364 | 0.652148 |
| Gen0_Mut0_ID1 | - | - | f | 0.74917 | 0.15789 | 0.610404 | 0.363917 |
| Gen0_Mut0_ID2 | - | - | m | 0.489214 | 0.479554 | 0.769761 | 0.386242 |
| Gen0_Mut0_ID3 | - | - | f | 0.389718 | 0.085979 | 0.176778 | 0.99077 |
| Gen0_Mut0_ID4 | - | - | m | 0.873439 | 0.908975 | 0.879219 | 0.899603 |
| Gen0_Mut0_ID5 | - | - | g | 0.374321 | 0.230493 | 0.129937 | 0.438298 |

## Gene Labelling and Hierarchy

*Note: The following is not necessary for all gene templates, only if you know the function of the labelled regions.*

For certain phenotypes you may find it to create hierarchical sections of the genome where multiple characters influence the same distinct trait.

For example, say you were using a genome which outputs an image with an RGB colour and has a pattern with a different RGB colour. You may wish for these colours to be able to mutate as a group and/or duplicate or mix between one another but not with other genes that influence other factors.

1. Group the genes in terms of function. I.e., position the genes for the background RGB and the pattern RGB together and in the same order.
2. Give the genes the same 1^st^ label e.g. Col = colour
3. Give the genes different 2^nd^ labels e.g. Bkg= background, Pat=pattern.
4. Make sure the R, G, B genes are in the same order.

| … | Col_Bkg_R | Col_Bkg_G | Col_Bkg_R | Col_Pat_R | Col_Pat_G | Col_Pat_B | … |
| --- | --- | --- | --- | --- | --- | --- | --- |
| … | 0.88 | 0.52 | 0.63 | 0.30 | 0.89 | 0.88 | … |
| … | 0.56 | 0.42 | 0.75 | 0.61 | 0.15 | 0.56 | … |

1. With the following gene arrangement R, G and B can be copied to the corresponding genes for background and pattern.

# Creating Your Own Fitness Calculators

Creating your own fitness calculator is relatively simple. All it requires is generating a script which can 1) convert the genes you created into the desired output; 2) measure fitness from the output and 3) create a ‘_survival.txt’ file for each generation in the folder where your population is stored. Example code for doing this can be found in the Travelling Salesperson and Background Match demo calculators located in the ImageGA plugin folder.

# File and Code Organisation

For the purposes of making ImageGA easier to edit, the code is organised as follows:

## /Fitness Calculators/:

Contains the .txt file for each fitness calculator.

- Background Match/: *Contains the background photo used by background match.*
- Salesperson/: *Contains the map and Gaussian Noise multiplier for the salesperson problems.*
- Population_Templates/: *Contains the .csv file templates for the genomes of each caclulator.*

## /RequisitFunctions/:

Contains the necessary plugins for running ImageGA.

- AlgorithmSettings.txt*: the previously used settings for ImageGA*
- Fitness_Plotter: *plots the change in fitness over time and concatenates the fitness values for each generation into a .csv file.*
- ImageGA_Algorithm: *The genetic algorithm component for ImageGA, will create a new generation within a destination folder providing that there is an AlgorithmSettings.txt file and a generation file within the assigned folder.*
- ImageGA_Interface: *The interface for adjusting the current ImageGA values and for creating generation_0 for a population.*
- PopulationLocation: *Records where ImageGA_Interface should create its new population and where the Fitness Calculators should save their fitness values to.*

## ImageGA Demo:

Acts as a demonstration for ImageGA combining the core functions together to allow users to run the genetic algorithm using the demo fitness calculators provided.

# Recommended Settings for Psychophysics Experiments

For small populations intended to be used for psychophysics or camouflage evolution experiments the following settings are recommended:

1. A population of at least 24individuals.
2. A deletion pool that does not remove the entire population.
3. A breeding pool that does not include all surviving individuals.
4. A random mating system.
5. A high mutation rate 0.01 – 0.1 for the non-breeding individuals if used.
6. A low mutation rate 0.001 – 0.005 for offspring.
7. Use higher rates for the lvl1 and lvl2 mutations.
8. Use random or multi_point crossover.
9. Allow for incomplete recombination.
10. Use targeted duplication for repeated genes e.g., colour for different patterns.
11. Use the mutant-mate crowd operator if you intend to run more than 10 generations.

This system ensures that the population isn’t too large for a single player to complete an entire generation; provides a buffer against noise killing off fit individuals by not removing too many; maintains diversity through random mating; ensures that offspring don’t undergo destructive levels of mutation while non-breeders are mutated to boost genetic diversity and provides methods for more rapidly optimising colouration.

# References

DERIGS, U., KABATH, M. & ZILS, M. 1999. Adaptive Genetic Algorithms: A Methodology for Dynamic Autoconfiguration of Genetic Search Algorithms. *In:* VOß, S., MARTELLO, S., OSMAN, I. H. & ROUCAIROL, C. (eds.) *Meta-Heuristics: Advances and Trends in Local Search Paradigms for Optimization.* Boston, MA: Springer US.

GOLDBERG, D. E. & HOLLAND, J. H. 1988. Genetic algorithms and machine learning.

JUNG, C., KIM, Y.-H., YOON, Y. & MOON, B.-R. 2016. A New Adaptive Hungarian Mating Scheme in Genetic Algorithms. *Discrete Dynamics in Nature and Society,* 2016**,** 3512546.

REJEB, J. & ABUELHAIJ, M. New gender genetic algorithm for solving graph partitioning problems. Proceedings of the 43rd IEEE Midwest Symposium on Circuits and Systems (Cat.No.CH37144), 8-11 Aug. 2000 2000. 444-446 vol.1.

YANG, S. Genetic Algorithms with Elitism-Based Immigrants for Changing Optimization Problems. *In:* GIACOBINI, M., ed. Applications of Evolutionary Computing, 2007// 2007 Berlin, Heidelberg. Springer Berlin Heidelberg, 627-636.
